# Reliable wafer-scale integration of two-dimensional materials and metal electrodes with van der Waals contacts

**DOI:** 10.1038/s41467-024-49058-7

**Published:** 2024-05-30

**Authors:** Xiaodong Zhang, Chenxi Huang, Zeyu Li, Jun Fu, Jiaran Tian, Zhuping Ouyang, Yuliang Yang, Xiang Shao, Yulei Han, Zhenhua Qiao, Hualing Zeng

**Affiliations:** 1 International Center for Quantum Design of Functional Materials (ICQD), Hefei National Research Center for Physical Sciences at the Microscale, University of Science and Technology of China, Hefei, China; 2 Hefei National Laboratory, University of Science and Technology of China, Hefei, China; 3 CAS Key Laboratory of Strongly Coupled Quantum Matter Physics, Department of Physics, University of Science and Technology of China, Hefei, Anhui China; 4 Department of Chemical Physics, University of Science and Technology of China, Hefei, Anhui China; 5 Department of Physics, Fuzhou University, Fuzhou, China

**Keywords:** Two-dimensional materials, Electronic devices

## Abstract

Since the first report on single-layer MoS_2_ based transistor, rapid progress has been achieved in two-dimensional (2D) material-based atomically thin electronics, providing an alternative approach to solve the bottleneck in silicon device miniaturization. In this scenario, reliable contact between the metal electrodes and the subnanometer-thick 2D materials becomes crucial in determining the device performance. Here, utilizing the quasi-van der Waals (vdW) epitaxy of metals on fluorophlogopite mica, we demonstrate an all-stacking method for the fabrication of 2D devices with high-quality vdW contacts by mechanically transferring pre-deposited metal electrodes. This technique is applicable for complex device integration with sizes up to the wafer scale and is also capable of tuning the electric characteristics of the interfacial junctions by transferring selective metals. Our results provide an efficient, scalable, and low-cost technique for 2D electronics, allowing high-density device integration as well as a handy tool for fundamental research in vdW materials.

## Abstract (web summary)

2D semiconductors may offer a platform for future electronics, but the wafer-scale fabrication of high-performance 2D transistors remains challenging. Here, the authors report a universal all-stacking method to fabricate wafer-scale 2D electronic devices with van der Waals contacts, based on epitaxial metallic electrodes grown on fluorophlogopite mica.

## Introduction

The emergence of two-dimensional (2D) semiconductors such as 2H-MoS_2_ holds promise for continuing Moore’s law since anticipated switching characteristics have been well demonstrated at the subnanometer channel scale^1–3^. Towards the ultimate 2D electronics, one critical issue is the realization of high-quality van der Waals (vdW) contact between the metal electrodes and the 2D materials in the device. However, if following the conventional device nanofabrication process in the modern semiconductor industry, this issue is rather challenging because vdW materials have an atomically thin film thickness that leads to serious metal/semiconductor interface problems such as Fermi-level pinning, unexpected doping, and even material damage during thermal metal deposition for making electrodes^4–8^. Therefore, there is the need to develop new methods that can overcome the interface contact problems in 2D semiconductor-based electronic devices^4,9–11^.

To tackle the above contact challenge in 2D material devices, several approaches have been recently developed, such as one-dimensional contact (or edge contact)^12–14^, low-temperature^11,15–19^ or buffer-layer assisted metal deposition^10,20–24^, and metal electrode mechanical transfer^4,25–34^. Among these methods, arbitrarily stacking the prefabricated metal electrodes onto the target 2D materials seems to be superior in device yields, technique sophistication, choice of metals, and interfacial Schottky barrier tunability. However, at the current stage, the metal transfer technique requires subtle modification of the silicon wafer either with the treatment of a specific passivator, such as hexamethyldisilazane (HMDS)^4,35^, or by covering a single layer of high-quality graphene to generate weak vdW interactions between the deposited metals and the substrate with reduced adhesion^27,29,36^, which might render its widespread application. Since the key in metal electrode transfer is the generation of the vdW interaction that allows the mechanical peeling of metal electrodes, it is natural to question whether there is a direct vdW growth approach of metals on modification-free substrates for transfer. With this advance, universal device fabrication for 2D electronics can be efficiently realized while maintaining the intrinsic electric transport characteristics.

Here, utilizing the quasi-vdW epitaxy of metals on fluorophlogopite mica (F-mica)^37^, we demonstrate that 2D electronic devices such as field-effect transistor (FET) and ferroelectric tunneling junction (FTJ) devices can be fabricated by simple peeling and stacking only. In our approach, metal electrodes with desired patterns were directly deposited on F-mica via a standard electron-beam evaporation process. Due to the high atomic crystallinity and mechanical strength of F-mica, the as-grown metal electrodes were mechanically exfoliated and arbitrarily transferred onto 2D semiconductors. The as-stacked 2D devices showed anticipated vdW interface contact, as evidenced by scanning transmission electron microscopy (STEM) imaging, and displayed advanced electric transport performance, including low off-state current, high switching ratio, and low contact resistance. By selecting different metal electrodes with compatible work functions, the interfacial Schottky barrier in the 2D device was optimized. We showed that the transfer of arbitrarily patterned metal electrodes from F-mica could be realized up to the wafer scale with yields close to 100%. Our results provide an all-stacking method for the universal fabrication of 2D material devices with the advantages of efficiency, reliability, and low cost.

## Results

### Intact transfer of patterned metals from F-mica

**Fig. 1:**
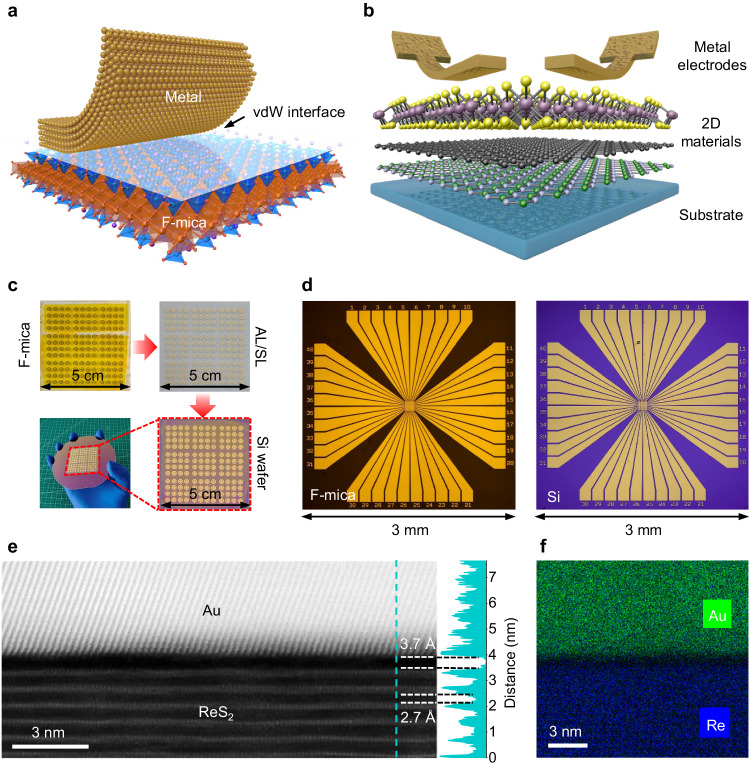
Illustration of electrode transfer and the all-stacking method. **a** Schematic diagram of the metal electrode peeling off process from fluorophlogopite mica (F-mica) with the van der Waals (vdW) interface. **b** Illustration of the all-stacking method. **c** Process of four square-inch electrode transfer, AL and SL represent adhesion layer and supporting layer respectively. **d** Optical images of the Au electrodes deposited on the F-mica (left) and transferred intact to the silicon substrate (right). **e** Cross-sectional STEM image of the stacked Au/ReS_2_ interface. The insert line profile (right) shows a vdW gap of ~3.7 Å at the ReS_2_/Au interface and ~2.7 Å at the ReS_2_/ReS_2_ interface. **f** The Au and Re elemental distribution at the cross-section of the stacked Au/ReS_2_ interface.

The key in our all-stacking method is to obtain freestanding and transferable metal electrodes. In this work, this is realized by utilizing the quasi-vdW epitaxial growth mechanism of most metals on layered F-mica. For quasi-vdW epitaxy, it has been generally accepted that materials grown on layered substrates interact weakly via the vdW force with the substrate^37^, other than by rigid chemical bonding, as in conventional lattice-matched crystal epitaxy. These weak vdW interactions provide the opportunity to easily separate the epilayer from the substrate. As seen in Fig. 1a, F-mica has a layered structure (Supplementary Fig. 1) that can be easily cleaved to produce an atomically flat surface (Supplementary Fig. 2) without dangling bonds, offering an appropriate substrate for quasi-vdW epitaxy^37^. The K^+^ ions layer on F-mica surface introduces the quasi-vdW interaction between metal epilayer and F-mica (Supplementary Fig. 3), resulting in quasi-vdW epitaxy of metal without the constraint on lattice match (Supplementary Fig. 4). In contrast to natural mica, F-mica is artificially synthesized with the replacement of (OH)^-^ ions by F^-^ ions in the lattice to obtain improved physical properties such as crystallinity and rigidity. The improved crystal stiffness guarantees that the epilayers, such as the thin metal films studied here, can be mechanically exfoliated by the “template stripping” method^38^ while maintaining a clean and atomically flat metal surface^39^. Based on these features, 2D electronic devices can be fabricated by directly stacking the transferable metal electrodes onto ultrathin 2D materials (see the schematic in Fig. 1b).

To prove the proposed all-stacking method, we first demonstrate the arbitrary transfer of patterned metal electrodes onto a silicon substrate with high uniformity at wafer scale. The transfer process for four square inches of electrode array is shown in Fig. 1c. Patterned metal (Au) electrodes are directly deposited on freshly cleaved F-mica by standard photolithography and electron-beam evaporation. An adhesion layer (AL) and a supporting layer (SL) are used to peel off and transfer all the metal electrodes from the F-mica. The hydrophilic property of F-mica assists the intactly peeling of electrodes^40^. Details of the process can be found in Methods, Supplementary Fig. 5-6. Benefiting from the weak vdW force at the interface, the exfoliated electrodes have an atomically flat and clean surface without the residual fragments or even the chemical components from F-mica, which can be evidenced from the energy-dispersive X-ray spectroscopy (EDS) in Supplementary Fig. 7, atomic force microscopy (AFM) in Supplementary Fig. 2, and the atomic-resolved interface image from STEM in Fig. 1e. Since the AL and SL are some kinds of polymers in nature, the as-exfoliated metal electrodes can then be transferred onto target substrates or materials in the same way as preparing and stacking 2D vdW materials. Here, after removing the AL and SL with heating and dissolving, the metal electrode was finally transferred onto a silicon substrate, as shown in Fig. 1c, d. From the optical images, we find that the transfer of patterned metal electrodes from the F-mica to the silicon substrate is almost intact (see Supplementary Fig. 8). Furthermore, we extended this method to many other metals, including silver (Ag), copper (Cu), and palladium (Pd), which are commonly used in 2D electronics of vdW materials. All these metals can be transferred with an almost 100% yield (see Supplementary Fig. 9). The compatibility of different metals might allow further tuning of the interfacial metal-semiconductor junction to optimize the electronic performance of 2D devices.

With the success of arbitrarily transferring metals, we next characterized the quality of the interface formed between the stacked electrodes and 2D materials. Here, ultrathin 1 T’-ReS_2_ was chosen as the representative of 2D semiconductors due to its high crystallinity, layer number independent semiconducting property^41^, and recently discovered sliding ferroelectricity^42^. The detailed crystal structure of 1 T’-ReS_2_ and sample preparation can be found in Supplementary Fig. 10 and Methods. On multilayer 1 T’-ReS_2_, we artificially stacked exfoliated Au electrodes to obtain a stacked 2D device. STEM was then used to visualize the metal-semiconductor interface. Figure 1e shows the cross-sectional STEM image of the interface between the stacked Au electrodes and 1 T’-ReS_2_. A clean gap of ~3.7 Å at the interface can be identified from Fig. 1e. The observed interfacial spacing is close to the physical vdW gap (defined as the chalcogen-to-chalcogen distance) of ~2.7 Å in bilayer or multilayer ReS_2_ (see the line profile in Fig. 1e). In addition, the distinctive element boundaries of Au and Re in Fig. 1f suggest no chemical doping for the ReS_2_ flake after stacking metal electrodes. These evidences indicate the formation of a vdW contact during the stacking process. Combined with the larger scale image in Supplementary Fig. 11 and all element mapping in Supplementary Fig. 12, an atomically sharp and clean interface without lattice disorder can be clearly seen, further confirming the reliable formation of the homogeneous vdW gap at the interface between the stacked Au electrodes and 2D materials.

### Electronic properties of the stacked 2D devices

The vdW contacts in the stacked 2D device were further verified by studying the electric transport behavior in FETs. To intuitively visualize the contact-related difference in device performance, we fabricated two types of FETs with stacked and deposited electrodes on the same flake of ultrathin 1 T’-ReS_2_. This also circumvents the effect of sample variation on the results. In the fabrication of FETs, ultrathin 1 T’-ReS_2_ was mechanically exfoliated onto a heavily doped Si substrate with 300 nm SiO_2_ on top. Doped Si was used as the global gate electrode, while SiO_2_ served as the gate dielectric. In all the devices, stacked metal electrodes were first used as the drain and source to characterize the intrinsic electronic performance to the greatest extent possible. After that, another pair of metal electrodes was deposited in situ on the same 2D semiconductor sample via standard lithography and electron-beam evaporation processes.

**Fig. 2:**
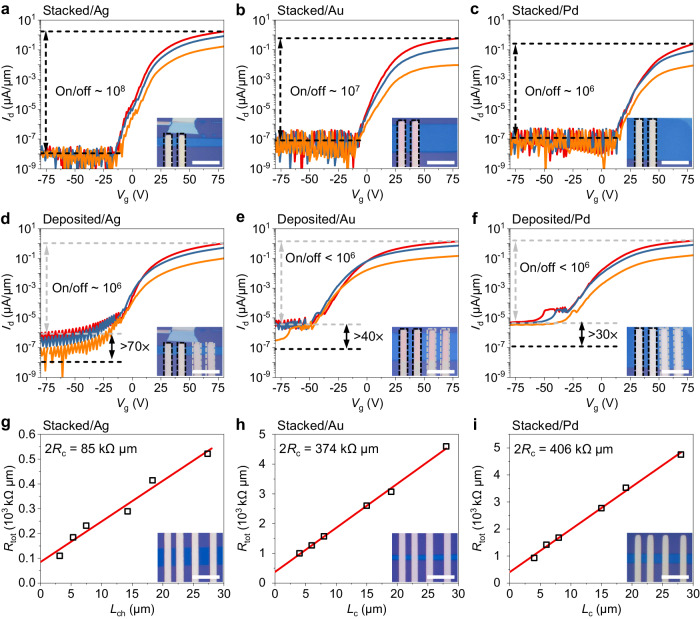
Electrical properties of ReS_2_ field effect transistors (FETs) with stacked and deposited metal electrodes. **a**–**c** Transfer characteristic curves of ReS_2_ FETs with Ag **a**, Au **b**, and Pd **c** electrodes by stacking. **d**–**f** Transfer characteristic curves of ReS_2_ FETs with Ag **d**, Au **e**, and Pd **f** electrodes by deposition. *I*_d_ and *V*_g_ represent the source-drain current and gate voltage, respectively. The red, blue and orange curves in **a**–**f** are measured under a drain voltage of 1, 0.5, and 0.1 V, respectively. The black dashed lines and gray dashed lines suggest the off-state current of FETs with stacked electrodes and deposited electrodes, respectively. Insets: Optical images of ReS_2_ FETs with stacked and deposited electrodes. The stacked electrodes and deposited electrodes are marked with black and gray dashed lines, respectively. **g**–**i** Contact resistance of ReS_2_ FETs with Ag **g**, Au **h**, and Pd **i** electrodes by stacking at *V*_g_ = 80 V. *R*_c_, *R*_tot_ and *L*_c_ represent the contact resistance, total resistance and length of the channel, respectively. Insets: Optical images of ReS_2_ FETs with stacked electrodes. The scale bar is 20 μm.

Figure 2 summarizes our main results on ultrathin 1 T’-ReS_2_-based FETs with different kinds of metal electrodes measured at room temperature under ambient conditions. For Ag-FETs, as shown in Fig. 2a, d, the device with stacked electrodes performs better than that with deposited electrodes in almost all indexes, such as the on/off ratio, subthreshold swing (*SS*), and electrical repeatability. In detail, owing to the vdW interface, the stacked Ag/ReS_2_ FET has a negligible off-state current (*I*_off_) below 10^−8^ μA/μm (Fig. 2a). In contrast, the deposited Ag/ReS_2_ FET has an approximately 70 times larger *I*_off_ of ~ 10^−6^ μA/μm (Fig. 2d) and is hardly switched off as a result of the damage to ReS_2_ due to “high-energy” metal deposition. As a whole, the stacked Ag/ReS_2_ FET has a higher *I*_on_/*I*_off_ ratio (~ 10^8^) and smaller *SS* (5.1 V/dec). The same enhancements in electronic performances can be seen in Au/ReS_2_ and Pd/ReS_2_ FETs (Fig. 2b, c, e, f), in which stacked FETs have smaller *I*_off_ and *SS* and higher *I*_on_/*I*_off_ ratios. It is notable that the stacked FETs show negligible hysteresis in the output characteristic curves, indicating that the interface of stacked electrodes/ReS_2_ is clean, whereas the defects from lithography and metal deposition result in larger hysteresis (see Supplementary Fig. 13). Similarly, the enhanced electronic performance of ultrathin WSe_2_-based stacked FETs with different metals can be found in Supplementary Fig. 14-16 and Supplementary Table 1.

Generally, if ignoring the defects and Fermi-level pinning effect, the contact resistance (*R*_c_) of the metal/semiconductor interface is proportional to ${e}^{{\phi }_{{{{{{\rm{B}}}}}}}}$ with ${\phi }_{{{{{{\rm{B}}}}}}}={\phi }_{{{{{{\rm{m}}}}}}}-\chi$ for an n-type semiconductor, where ${\phi }_{{{{{{\rm{B}}}}}}}$ is the Schottky barrier height, ${\phi }_{{{{{{\rm{m}}}}}}}$ is the work function of the metal, and $\chi$ is the electron affinity of the semiconductor. Therefore, by stacking different metal electrodes with varied ${\phi }_{{{{{{\rm{m}}}}}}}$ in multilayer ReS_2_-based FETs, the interfacial *R*_c_ can be optimized. Figure 2g–i shows the measured *R*_c_ in ReS_2_-based FETs with stacked Ag, Au, and Pd electrodes via the transfer length method (detailed in Methods)^10,16,27^. As anticipated, varied *R*_c_ was observed, which follows the same trend of variation in *I*_on_ as shown in Fig. 2a–c. For ReS_2_-based stacked FETs, Ag electrodes were found to provide the lowest *R*_c_ at 42.5 kΩ μm among all the different metals used in this study. This result also indicates a smaller ${\phi }_{{{{{{\rm{B}}}}}}}$ between 2D ReS_2_ and Ag than Au, Pd, and Cu. The optimization of ${\phi }_{{{{{{\rm{B}}}}}}}$ was further confirmed by the surface potential images of the stacked devices measured by Kelvin probe force microscopy (KPFM) in air, as shown in Supplementary Fig. 17. For different metals stacked on the same ReS_2_ multilayers, the contact potential difference between Ag and ReS_2_ was found to be the smallest at ~ 99.7 mV, which is consistent with the result from our *R*_c_ measurements. For an intuitive comparison, the performances of stacked ReS_2_ FETs with different metal electrodes are summarized in Table 1 (see Supplementary Fig. 18).

**Table 1:** Summary of the electronic performance of stacked FETs based on multilayer ReS_2_ with various metal electrodes

	Ag	Cu	Au	Pd
*I*_on_ (μA/μm)	1.73	0.086	0.6	0.25
*I*_on_/*I*_off_	10^8^	10^6^	10^7^	10^6^
*R*_c_ (kΩ μm)	42.5	2729.5	187	203
*V*_T_ (V)	32.2	46.3	27.4	54.0
*SS* (V/dec)	4.4	6.9	5.0	5.5

*V*_d_ = 1 V, *L*_c_ = 5 μm, and *R*_c_ are extracted at *V*_g_ = 80 V. *I*_on_ on-state current; *I*_off_ off-state current; *R*_c_ contact resistance; *V*_T_ threshold voltage; *SS* subthreshold swing.

For the carrier mobility, we found negligible improvement in the stacked FETs if compared with the devices using deposited electrodes. For instance, from the data in Fig. 2a, the low-field field effect mobility of the stacked Ag/ReS_2_ FET is extracted to be 15.04 cm^2^ V^−1^ s^−1^ via *μ* = *gL*_c_/(*W*_c_*C*_ins_*V*_d_), where *g* is the transconductance, *L*_c_ and *W*_c_ are the channel length and width, *C*_ins_ is the gate insulator capacitance, and *V*_d_ is the source-drain voltage. This value is marginally better than that measured in deposited Ag/ReS_2_ FET at 8.96 cm^2^ V^−1^ s^−1^. The carrier mobility in FETs is mainly determined by the dielectric environment and the defects density of the channel material^43,44^. Therefore, the same gating dielectric and the identical quality of ultrathin 1 T’-ReS_2_ used in the stacked and deposited FETs result in the similar carrier mobilities.

### Massive integration of 2D devices via stacking

**Fig. 3:**
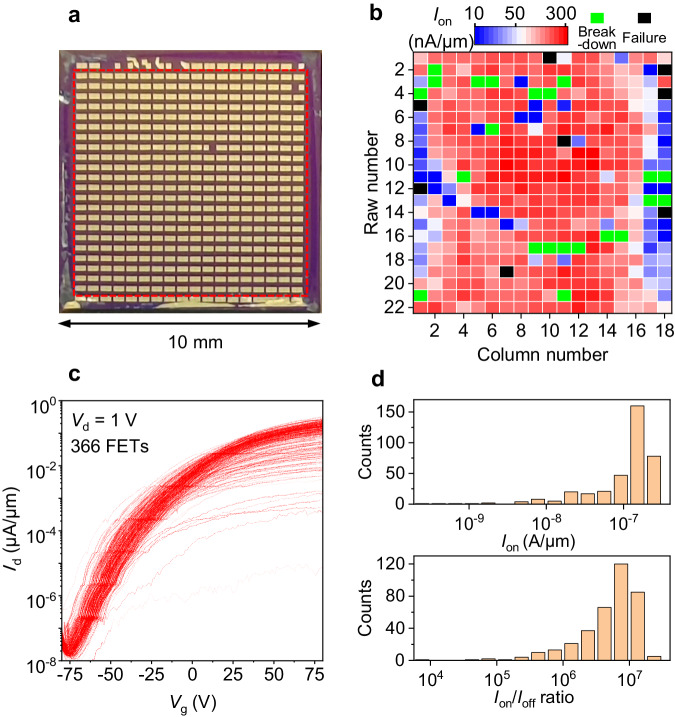
Electronic performances of the 10 × 10 mm^2^ ML MoS_2_ FET array. **a** Optical image of the ML MoS_2_ FET array with stacking Ag/Au electrodes on SiO_2_/Si. **b** Mapping of the *I*_on_ from the 396 ML MoS_2_ FETs shown in the red dashed box in **a**. **c** Transfer characteristic curves of the 366 FETs shown in **b**, excluding 22 breakdown FETs and 8 failed FETs. **d** Histogram of the *I*_on_ and *I*_on_/*I*_off_ ratios extracted from the 366 transfer characteristic curves in **c**.

To demonstrate the potential wafer-scale capability of our method, we showed that massive FET devices can be fabricated by using chemical vapor deposition (CVD) synthesized large-scale monolayer (ML) MoS_2_. Figure 3a displays a ML MoS_2_ FET array with a scale up to 10 × 10 mm^2^ fabricated by the all-stacking method. Details of the fabrication process can be found in Methods and Supplementary Fig. 19. From the optical image in Supplementary Fig. 19c, we found 416 intact FET devices, equaling to a device yield of 416/432 ≈ 96.3%. The broken FETs might be due to the residues from the etching process of the ML MoS_2_ array and the slicing induced substrate damage at edges.

The electronic performances of the stacked MoS_2_ FET array were further studied. In the measurements, 22 devices at the top and the bottom rows were excluded due to the potential leakage from damaged silicon substrate. The 394 ML MoS_2_/Ag FETs studied are highlighted with red dash lines in Fig. 3a. Their *I*_on_ are summarized in Fig. 3b. In these FETs, we only found 6 devices not functioning. The failure of 6 FETs can be partly attributed to the damage of MoS_2_ channel ribbons during etching^45^. Therefore, the yield of our method in producing functioning FETs is (394-6)/394 ≈ 98.4%. In measuring the transfer characteristic curves, 22 FETs were breakdown during test, which is due to the defects in SiO_2_. As shown in Fig. 3c, d, the as-fabricated 366 2D FETs exhibit highly uniform electronic performances, whose average *I*_on_ and on/off ratio are 140 nA/μm and 6.8 × 10^6^, respectively. Among the 366 FETs, the *I*_on_ is larger than 0.1 μA in 97.5% of all the devices, and is larger than 1 μA in 82.0% FETs. For the *I*_on_/*I*_off_ ratio, it is found to be larger than 10^6^ in 91.3% of all the devices. For a comparison, the electronic performances of stacked ML MoS_2_ FETs studied here and those in previous works are summarized in Supplementary Table 2, showing the high reliability of our all-stacking method at large-scale.

### Vertical integration of 2D devices via multiple stacking

**Fig. 4:**
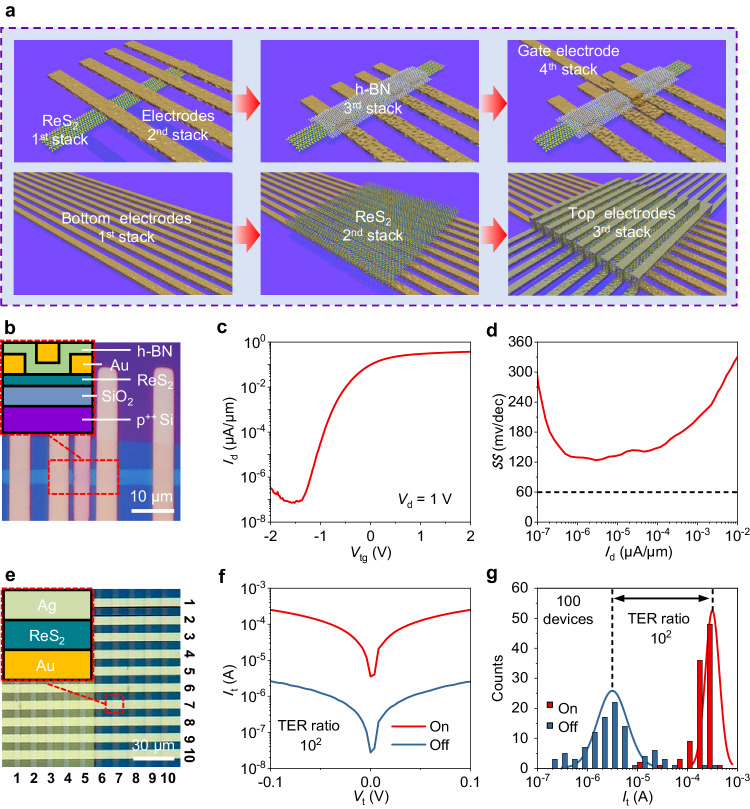
All-stacking 2D devices with multiple layer structure. **a** Schematics of the integration processes of vertical multiple layer devices by the all-stacking method. **b** Optical image of the all-stacked ReS_2_/Au FET in Fig. 2h. Gate dielectric/top gate fabricated by transferring the h-BN/Au layer. Inset: cross-sectional schematic of the red dashed line area. **c** Transfer characteristic curves of the ReS_2_ FET in **b**. *V*_tg_ represents the top-gate voltage. **d** Subthreshold swing (*SS*) at different current densities of the ReS_2_ FET in **b**. The dashed line represents theoretical limit of *SS* at room temperature, 60 mV per decade. **e** Optical image of the all-stacked Ag/ReS_2_/Au FTJ array. Inset: the cross-sectional schematic of the red dashed line area. **f** *I*–*V* characteristics of a ReS_2_ FTJ device exhibiting a tunneling electroresistance (TER) ratio ~ 10^2^. *I*_t_ and *V*_t_ represent the tunneling current and tunneling voltage, respectively. **g** Histogram of the off state and on state tunneling current of the 100 ReS_2_ FTJs in **e**. The two vertical dashed lines indicate the mean of the fitted normal distribution curves (red and blue solid lines) of the off and on currents. The TER ratio between the averaged on and off current is ~ 10^2^.

We next show that the all-stacking method can be seamlessly applied to fabricate 2D devices with vertical multiple layer structures. In 2D vertical electronic devices, at least a pair of bottom and top metal electrodes is required to sandwich the ultrathin vdW materials. Conventionally, repeated processes of photolithography and thermal metal deposition are utilized, which inevitably results in the abovementioned interfacial contact issue and increases the device fabrication complexity. With transferable metal electrodes, such a structure can be realized conveniently by stacking. Here, we demonstrate two typical types of stacked vertical 2D devices, namely, the top gate FET device and the FTJ device, that might be of broad interest for the study of 2D electronics. Figure 4a shows the multiple integration processes of metal electrodes in vertical 2D devices. For assembling multilayer ReS_2_-based top gate FETs, the bottom and top metal electrodes are selectively transferred onto multilayer ReS_2_ at separated 2^nd^ and 4^th^ stacking steps to serve as the drain/source and top gate electrodes, respectively, and the insulating h-BN layers are transferred at the 3^rd^ stacking step to serve as the gate dielectric (see the upper panel in Fig. 4a). For fabricating the FTJ device, a sandwich-like structure is realized by placing different metal electrodes via 1^st^ and 3^rd^ stacking steps (see the lower panel in Fig. 4a).

Figure 4b presents the optical image of the as-stacked 4-layer ReS_2_-based top gate FET device. It should be noted that this top-gate FET device is based on the stacked Au/ReS_2_ FET, as shown in Fig. 2h. After the electrical measurements with the bottom global gate, a few layers of h-BN and the Au electrode were stacked onto the Au/ReS_2_ FET to serve as the gate dielectric and top gate, respectively. More details can be found in Supplementary Fig. 20a. As shown in Fig. 4b–d, the device remained intact and still functioned well with a high on/off ratio (~10^7^) after these multiple overlay stacking processes. Furthermore, we found the enhanced *SS* to be 120 mV/dec (Fig. 4d) in the device compared to the result via bottom global gating at 5.0 V/dec, as shown in Supplementary Fig. 21. The enhanced *SS* originates from the reduced equivalent oxide thickness of the top gate dielectrics.

For stacked FTJs, we show that an array of 100 Ag/ReS_2_/Au FTJ devices (Fig. 4e) can be simultaneously fabricated via the all-stacking method. The detailed integration process of the FTJ device array can be found in Supplementary Fig. 20b. We observed a distinct on/off switch from the FTJs by applying external poling voltages. Figure 4f displays the characteristic I–V measurements at on/off states from one typical FTJ device. The tunneling current of the on and off states under 0.1 V reading bias were found to be ~100 μA and ~1 μA, showing a tunneling electroresistance (TER) ratio at ~ 10^2^. The switch in TER can be understood as the electrical control of the out-of-plane sliding ferroelectricity in multilayer 1 T’-ReS_2_^42^. Moreover, we observed different initial TERs in the 100 FTJ devices, which are summarized in Fig. 4g and Supplementary Fig. 22. By statistics, the initial TERs were categorized into two types that correspond to the on and off states. It is worth pointing out that the statistics on the initial states also yield an on/off ratio at ~ 10^2^, which is consistent with the result in Fig. 4f via external electric field poling. The observed random distribution of the initial TERs is due to the spontaneous electric polarizations in multilayer 1 T’-ReS_2_, which is one characteristic feature of ferroelectrics.

### Modeling of the vdW interactions between metals and F-mica

The demonstrated all-stacking method essentially relies on the weak interfacial interactions between metals and layered F-mica due to quasi-vdW epitaxy. We show that this interfacial effect can be theoretically interpreted. To quantitatively study the weak interaction or adhesion between metals and F-mica, we use the projected-augmented-wave method as implemented in the VASP package^46^ and generalized gradient approximation exchange correlation potential^47^ in the calculations. Here, the DFT-D3 method is adopted to describe the vdW force in the metal/F-mica heterostructure^48^. In the heterostructure, the (001) crystal face of F-mica is cleaved to support different deposited metals. To underline the effect of quasi-vdW epitaxial growth on F-mica, a SiO_2_ substrate^49^ is used as the control group substrate. To estimate the exfoliation feasibility, the exfoliation energy is defined as *E*_ex_ = (*E*_metal_ + *E*_sub_ − *E*_hetero_)/*A*, where *E*_metal_, *E*_sub_, and *E*_hetero_ are the energies of the metal, substrate and metal/substrate heterojunction, and *A* stands for the in-plane area of the surface unit cell.

**Fig. 5:**
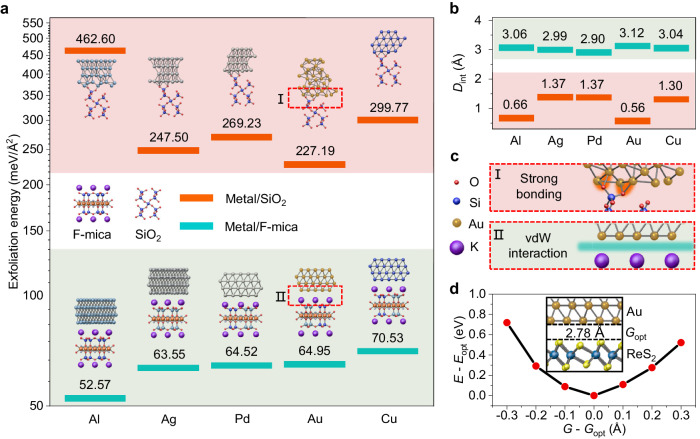
The theoretical calculation of the interface state between the metal and support substrates. **a** Exfoliation energy for peeling deposited Al, Ag, Pd, Au and Cu layers from F-mica and SiO_2_. Insets are schematic diagrams of the interaction between various metals (Al, Ag, Pd, Au and Cu) on F-mica and SiO_2_ substrates after relaxation. **b** The interfacial distance (*D*_int_) between different metal atoms and substrates. The cyan lines and the orange lines represent metal/F-mica and metal/SiO_2_, respectively. **c** Enlarged schematic of Au atoms on F-mica and SiO_2_ substrate interactions in areas I and II in **a**. **d** The relationship between the gap and the energy of the stacked Au/ReS_2_*. G* (*G*_opt_) and *E* (*E*_opt_) represent the gap (optimal gap) of the stacked Au/ReS_2_ and the energy (optimal energy) of the whole stack structure, respectively. The *G*_opt_ is 2.78 Å. The inset is a schematic diagram of the stacked Au/ReS_2_.

The calculated exfoliation energies for different metals, including Al, Ag, Pd, Au, and Cu, from F-mica and SiO_2_ are summarized in Fig. 5a. Generally, the calculation result suggests a much lower *E*_ex_ of metals on F-mica than that for the same metal on SiO_2_. Among these metals, Al is found to have the smallest exfoliation energy. Furthermore, as shown in Fig. 5b, after full relaxation, the interfacial distance *D*_int_, ranges from 2.90 Å to 3.12 Å for different metal/F-mica heterostructures, which meets the representative range of vdW gaps. As a comparison, shorter *D*_int_ values ranging from 0.56 Å to 1.37 Å are found in the heterostructure of metals and SiO_2_. From these results, we can safely exclude the formation of chemical bonds between the deposited metals and F-mica, which confirms the quasi-vdW epitaxy, while we find strong bonding between the deposited metal films and SiO_2_ substrate due to the surface dangling bonds in SiO_2_ (Fig. 5c). These results in turn explain the smaller *E*_ex_ of metals found on F-mica than that on SiO_2_. Together with the formation of vdW gaps, the small *E*_ex_ guarantees that the intact metal electrodes peel from the F-mica, which contributes to the realization of our all-stacking method.

Furthermore, in the stacked 2D devices, we found that the vdW gap (~3.7 Å) of the Au/ReS_2_ interface is slightly larger than the natural vdW gap (~2.7 Å) in multilayer ReS_2,_ as shown in the STEM measurements (Fig. 1e). However, by studying the energy of the whole stacked heterostructure as a function of the interfacial gap (*G*), we found the optimal gap (*G*_opt_) to be 2.78 Å (Fig. 5d). The calculated *G*_opt_ is exactly close to that in multilayer ReS_2_. The deviation in experimental observation suggests that the interfacial gap distance in the stacked devices can be further improved via methods such as postannealing. Reducing the vdW gap can help to eliminate additional tunneling resistance at the interface, offering enhanced device performance with optimized *R*_c_^11^ that contributes to revealing the intrinsic electronic properties of 2D semiconductors.

## Discussion

In summary, we demonstrate a reliable all-stacking method for universal 2D device integration with optimized interfacial vdW contacts that can be extended up to the wafer scale. The stacking established vdW contacts between 2D materials and different metals benefit the optimization of interfacial Schottky barrier heights in the devices. For 2D devices with multiple layer structures, the all-stacking method increases the device fabrication efficiency and yields that can lower the threshold to demonstrate promising applications based on 2D materials with massive devices. Our results provide a fresh concept for future industrial integration of 2D materials, allowing not only high-density device integration but also the feasibility of studying the intrinsic transport properties of vdW materials.

## Methods

### Metal electrode fabrication, peeling and stacking

All metal electrodes for transferring are constructed on freshly cleaved F-mica using standard lithography and electron-beam evaporation technology. After electrode fabrication, poly(methyl methacrylate) (PMMA) solution (950 4 A) is spin-coated on top of the electrodes/F-mica as an adhesion layer. Then, the support layer, including the polydimethylsiloxane (PDMS) film and glass sheet, further covers the top of the adhesion layer and is heated to 50 °C for 10 min. The fabricated electrodes are peeled off smoothly after the whole stack sample is soaked in water overnight. In rapid sequence, the electrodes are laminated to the target substrate via the mechanical aligner with an optical microscope. Along with the lamination process, the whole stack sample is heated to 150 °C for 5 min. Finally, the support layer is peeled off, and the adhesion layer is dissolved in acetone for 5 minutes, leaving the electrodes on the target substrate. All electrode peeling and stacking operations are performed under ambient conditions.

All metal electrodes for electrical measurement include 20 nm contact metals (Ag, Cu, Au and Pd) and 40 nm Au. Each metal electrode for KPFM consists of only a single type of metal (Ag, Au and Pd).

### 2D material fabrication

The ReS_2_, WSe_2_ and h-BN flakes are mechanically exfoliated and transferred to a 300-nm SiO_2_ substrate using the all-dry transfer technique^50^. The ReS_2_ crystals are synthesized using the chemical vapor transport (CVT) method with I_2_ as the transport agent^42^. WSe_2_ and h-BN crystals are commercial products (HQ Graphene Co.).

### Fabrication of the stacked 2D FET array

The stacking process of fabricating massive 2D device is summarized in Supplementary Fig. 19. Here, the CVD ML MoS_2_ on 300 nm SiO_2_ substrate is used as the channel material (commercially available from Sixcarbon Tech. Shenzhen Co.). By standard lithography and dry etching, the large-scale CVD ML MoS_2_ is spatially patterned into 500 μm × 400 μm separated ribbons. Each ribbon has a length of 60 μm and a width of 20 μm (see Supplementary Fig. 19d). Accordingly, an array of metal electrodes patterned in 24 rows and 18 columns is then transferred onto the CVD ML MoS_2_. The support layer is peeled off and the adhesion layer is dissolved once the electrodes is stacked onto the SiO_2_ substrate (see Supplementary Fig. 19a–c). The source/drain electrodes consist of Ag (20 nm) and Au (40 nm) because the *R*_c_ of MoS_2_/Ag is smallest among common metals^4,27^. The Au layer serves to protect the Ag from oxidation.

### Electrical measurement

All electrical measurements are performed with a Keithley 4200 A semiconductor characterization system in a probe station at room temperature under ambient conditions. *R*_c_ is extracted through the transfer-length method. The total resistance (*R*_tot_) of the ReS_2_ FET can be expressed as *R*_tot_ = 2*R*_c_ + *R*_ch_ = 2*R*_c_ + *R*_sh_*L*_ch_, where *R*_c_ is the contact resistance, *R*_ch_ is the channel resistance, *R*_sh_ is the sheet resistance of the ReS_2_ channel and *L*_ch_ is the channel length. *R*_c_ can be extracted from the vertical intercept of the *R*_tot_-*L*_ch_ graph by measuring the *R*_tot_ of devices with various *L*_ch_.

### Other characterizations

The AFM measurements are performed on a Dimension Icon (Bruker Co.) with commercial silicon tips (VTESPA-300, Bruker Co.). In particular, the KPFM image is measured using doped silicon tips with a metal coating (SCM-PIT-V2, Bruker Co.). Scanning electron microscopy (SEM) and EDS measurements are performed using a GeminiSEM 500 (Zeiss Co.) with an energy-dispersive X-ray spectrometer (Aztec, Oxford Co.). The cross-sectional STEM images are taken by a Titan Themis-Z TEM with a probe corrector (Thermo Fisher Scientific Co.).

### Details of calculation

We use the projected-augmented-wave method as implemented in the VASP package^46^ and generalized gradient approximation exchange correlation potential in the calculations^47^. The kinetic cut-off energy of the plane wave is set to 400 eV. The Brillouin zone is sampled with a Gamma-centered grid with a k-point density of 2π × 0.02 Å^−1^ based on the scheme proposed by Monkhorst-Pack for calculation of structural optimization and self-consistent calculations^51^. A vacuum buffer space over 18 Å is included to prevent interaction between adjacent slabs. The convergence criterion is set to 10^−5 ^eV for energy in optimization and self-consistent calculations, respectively. During structural optimization, the lattice constants of metal films are used, and all atoms are fully relaxed except for the six layers of atoms of F-mica at the bottom. The Hellmann-Feynman force tolerance criterion for convergence is 0.01 eV/Å. The DFT-D3 method is adopted to describe the van der Waals force in the metal/F-mica heterostructure^46^. The (001) face of F-mica is the surface to support different deposited metals. For Au, Cu and Pd, 2 × 2 supercells are used to match the F-mica. However, for Ag and Al, 2$\sqrt{7}$ × 2$\sqrt{7}$ supercells are used to match the F-mica. On F-mica, the lattice mismatches are 5.11%, 5.56%, 4.78%, 3.66% and 3.69% for Al, Ag, Pd, Au and Cu, respectively. α-SiO_2_ is used as the control group substrate. For F-mica, the exfoliation energies are 52.57, 63.55, 64.52, 64.95, and 70.53 meV/Å^2^ for Al, Ag, Pd, Au and Cu, respectively. The interfacial distance between deposited metals and F-mica are 3.06, 2.99, 2.90, 3.12 and 3.04 Å for Al, Ag, Pd, Au and Cu, respectively. For α-SiO_2_, the exfoliation energies are 462.60, 247.50, 269.23, 227.19 and 299.77 meV/Å^2^ for Al, Ag, Pd, Au and Cu, respectively. The interfacial distance between deposited metals and α-SiO_2_ are 0.66, 1.37, 1.37, 0.56 and 1.30 Å for Al, Ag, Pd, Au and Cu, respectively. To obtain the optimal gap between 1 T’-ReS_2_ and Au film, the Au atoms are fixed during structural optimization.

### Supplementary information

Supplementary Information

Peer Review File

## Data Availability

The data that support the findings of this study are available from the corresponding author on request.
