# Supplementary material for: Reliable wafer-scale integration of two-dimensional materials and metal electrodes with van der Waals contacts
Source: Nat Commun. 2024 May 30;15:4619. doi: 10.1038/s41467-024-49058-7 (PMC11139895; doi:10.1038/s41467-024-49058-7)
Supplement: Supplementary file 1 — Supplementary Information [file 41467_2024_49058_MOESM1_ESM.pdf]

Supplementary Information for

"Reliable wafer-scale integration of two-dimensional materials and metal electrodes with van der Waals contacts"

Xiaodong Zhang[1,2,3, #], Chenxi Huang[1,3,4 #], Zeyu Li[1,2,3, #], Jun Fu[1,2,3], Jiaran Tian[2,3], Ouyang Zhuping[2,3], Yuliang Yang[2,3], Xiang Shao[4], Yulei Han[5], Zhenhua Qiao[1,2,3], and Hualing Zeng[1,2,3, *]

[1]*International Center for Quantum Design of Functional Materials (ICQD), Hefei National Research Center for Physical Sciences at the Microscale, University of Science and Technology of China, Hefei 230026, China.*

[2]*Hefei National Laboratory, University of Science and Technology of China, Hefei 230088, China.*

[3]*CAS Key Laboratory of Strongly Coupled Quantum Matter Physics, Department of Physics, University of Science and Technology of China, Hefei, Anhui 230026, China.*

[4]*Department of Chemical Physics, University of Science and Technology of China, Hefei, Anhui 230026, China.*

[5]*Department of Physics, Fuzhou University, Fuzhou 350108, China.*

* Corresponding author: hlzeng@ustc.edu.cn

\# Contribute equally to this work

Supplementary Notes

Supplementary Note 1.: The electrodes transferring.

All metal electrodes are fabricated on freshly cleaved F-mica by standard lithography and electron-beam evaporation processes. All electrodes for electrical measurement are 60 nm thick including 20 nm contact metals (Ag, Cu, Au and Pd) and 40 nm Au to prevent oxidation. The typical time of the lift-off process is 45 minutes via N-methyl-2-pyrrolidone (NMP). To peel off electrodes, poly(methyl methacrylate) (PMMA) solution (950 4A) is spin-coated on the top of the electrodes/F-mica at 4000 rpm for 40 seconds as the adhesion layer (AL). After cooling to room temperature, a piece of polydimethylsiloxane (PDMS) and glass sheet cover the top of the AL as the support layer (SL) and then heated at 50 °C for 10 minutes for better adhesion. For easy peeling, the whole stack sample is soaked in water overnight to create a tiny gap between the AL and the F-mica at the edges owning to the hydrophilic nature of F-mica[1]. Otherwise, the SL/AL stack will be firmly pressed against the F-mica due to the atmospheric pressure and difficult to be peeled off. The SL/AL/electrodes stack can be easily peeled from the F-mica after taking the whole stack out of the water and blowing the water out with nitrogen gas. The bottom surface of metal electrodes is clean throughout the whole peeling process, because the water does not dip into the interface of AL/F-mica even after the stack has been soaked in water for three days.

All electrodes are laminated to the target substrate via the mechanical aligner under an optical microscope due to the transparency of the AL and the SL. Along with the lamination process, the whole stack is heated to 150 °C for 5 minutes to make strong adhesion between AL/electrodes and the target substrate. Finally, the SL is peeled off and the AL is dissolved in acetone for 5 minutes, leaving the electrodes on the target substrate. All electrodes peeling and stacking operation are performed under ambient conditions.

Supplementary Note 2: The vertical fabrication of multilayer 2D devices.

4-layer ReS_2 FET: First, a piece of ReS_2 flake is mechanically exfoliated and transferred via PDMS[2] onto a heavily doped silicon substrate (as gate) with 300-nm silicon oxide (as gate dielectric). Then, source and drain electrodes are transferred onto the ReS_2 flake via the method in Note 1. After removing the SL and AL, a piece of h-BN is transferred onto the electrodes/ReS_2 stack. Finally, an electrode is transferred on the top of h-BN as gate electrode.

3-layer ReS_2 FTJ array: First, 10 electrodes are transferred onto a SiO_2 substrate as the bottom electrodes. Then, a piece of ReS_2 flake is mechanically exfoliated and transferred via PDMS onto the bottom electrodes as the tunneling layer. Finally, the other 10 electrodes are transferred onto the ReS_2 flake as the top electrodes.

All transferring operations take place at the mechanical aligner under an optical microscope, achieving alignment between layers.

Supplementary Note 3: Synthesis of ReS_2 crystals.

High-quality ReS_2 crystals are synthesized using the Chemical Vapor Transport (CVT) method with I_2 as the transport agent[3]. Typically, Re (99.99%, Aladdin Co.) powder and S (99.998%, Macklin Co.) powder are sealed in a quartz ampoule under vacuum with a 1:2 ratio. The ampoule is put into a two-zone furnace with a temperature gradient of 980 °C to 1080 °C. After 2 weeks, the ampoule is cooled down to room temperature in 48 hours. The crystals are annealed at 300°C for 24 hours to remove excess sulfur and iodine.

Supplementary Note 4: Electronic properties of the stacked WSe_2-based FETs.

The enhanced electronic performance of ultrathin WSe_2-based stacked FETs with different metals are summarized in Supplementary Figure 14-16. As shown in Supplementary Figure 14, after stacking different metal electrodes, the WSe_2-based FETs show a transition from p-type character to ambipolar character when the stacked electrodes change from Pd to Au or Ag. In contrast, with deposited electrodes, WSe_2-

based FETs trend to exhibit ambipolar or n-type behavior. Especially, for Pd electrodes, the as fabricated FET suffers device failure when using the thermal deposition method (see Supplementary Figure 14c, 14f, and Supplementary Figure 15).

Similar to ReS_2-based FETs, higher channel currents are observed in stacked WSe_2-based FETs if comparing with the devices with deposited electrodes[4,5]. In addition, the output characteristic curves show almost negligible hysteresis in the stacked devices, suggesting that the interface contact is clean between the 2D semiconductor and stacked electrodes. Supplementary Table 1 summarizes the electrical performance of stacked ML WSe_2-based FET (p-type) with that in previous works. The as- fabricated ML WSe_2-FET by F-mica-assisted all-stacking method performs enhanced on current and on/off ratio.

Supplementary Note 5: Discussions on the high yield of the all-stacking method.

The high successful rate in metal electrode transfer is what we want to demonstrate in this work. The nearly 100% yield of our 2D all-stacking method is attributed to the following aspects.

The unique structure of layered F-mica accounts for the primary reason. As a quasi-vdW material, F-mica has a layer structure with an inactive surface, as shown in Supplementary Figure 1. Therefore, F-mica has been used as the quasi-vdW epitaxy substrate to grow epitaxial overlayers, such as oxides, 2D materials, and even metals[6]. We observed clear layered structure with quasi-vdW interactions between layers as mediated by intercalated K ions. Our result is in consistence with that in previous studies[6]. The quasi-vdW interaction and the inactive surface of F-mica guarantee the easy exfoliation of the crystal itself and the epilayers.

The second reason is the quasi-vdW epitaxy of metals on layered F-mica. After depositing metals onto F-mica, high-quality crystalline metals are formed without the constraint on lattice match as shown in Supplementary Figure 4. These atomic-resolved

cross-sectional STEM images clearly showed the quasi-vdW epitaxy mechanism. The interface between metal epilayer and F-mica follows the quasi-vdW interaction with the intercalation of K ions (see Supplementary Figure 3). Therefore, when using the mechanical exfoliation method, the metal electrodes can be intactly peeled off for transfer as shown in Fig. 1a. Moreover, the EDS results (see Supplementary Figure 7) indicated that there are no residuals from F-mica on the surface of exfoliated metal electrodes, which further proves that metal epilayers and the F-mica substrate are weakly interacted via vdW force.

Third, the successful rate of metal transfer at large-scale is attributed to the peeling technique developed in this work. Due to the flexibility of AL, the AL/F-mica fits perfectly after spin-coating. The SL/AL stack will be firmly pressed against the F-mica due to the atmospheric pressure. Therefore, if directly peeling the AL/SL from F-mica, the applied force will distribute unevenly across the entire AL/SL layer, resulting in partial electrode damage during the transfer process. To overcome this issue, we utilized the hydrophilic nature of F-mica. The whole stack sample is soaked in water overnight to create a tiny gap between the AL and the F-mica at the edges. With longer soaking time, the gap becomes bigger until the AL/F-mica are completely separated. According to our experience, most of the AL/F-mica separate completely in water after soaking for more than 7 days. Therefore, before the complete separation that will lead to water contamination on the lower surface of the electrodes, the AL/F-mica was normally taken out after soaking overnight with a tiny gap at the edge for easy exfoliation. This soaking-assisted peeling technique help to intactly transfer the metal electrodes as depicted in Supplementary Figure 5a.

Supplementary Note 6: Technical potential of the all-stacking method.

Compared to previous methods of realizing the ideal vdW contact between the metal electrodes and the 2D materials in the device[4,5,7-10], there are two main superiorities in

technical potentials of F-mica-assisted all-stacking technique. The superiorities and the industrial potential of our method are discussed in the following.

The first is the low cost for fabrication of 2D materials-based devices at large-scale. In our all-stacking method, complex pretreatments of the substrates with coatings such as HDMS or high-quality single layer graphene substrate can be avoided. If taking the cost of complex pretreatments on silicon or the price of monolayer graphene into account, F-mica assisted mechanical electrode transfer seems to be a more cost-effective and efficient approach. Additionally, the transfer of complex electrodes from F-mica can be done in most labs even if the chemical operations are not permitted or conditioned. Sophisticated processes that require a significant amount of labor and time can be saved. Therefore, it lowers the threshold for the preparation of effective 2D devices, and enables colleagues and peers from diverse disciplines to study the rich fundamental properties of 2D materials. Furthermore, F-mica has a layered structure, which enables multiple times of repeated use by surface cleavage. To deal with larger wafers in industry, the developed multiple stacking approach might provide a solution. For example, in Fig. 1c, patterned metal electrodes at 2-inch scale have been intactly transferred onto a 4-inch silicon wafer. If repeatedly the stacking, the 4-inch silicon wafer can be fulfilled. With the above features, we thus emphasize the low cost of our method in the manuscript. If being adopted in the industry, these features might speed up the manufacturing process in high-density devices, and enables rapid and batch production.

The second benefit of our all-stacking method is the improved device performances. For individual device, stacked 2D FET performs better than that with deposited electrodes in almost all indexes, such as the on/off ratio, subthreshold swing (*SS*), and electrical repeatability as shown in Fig. 2, Supplementary Figure 13-16, and Supplementary Table 1. For massive devices at large scale, we show that they can be fabricated by our method when using CVD synthesized large-scale ML MoS_2 as shown in Fig. 3. The as-fabricated 366 2D FETs exhibit highly uniform electronic

performances. A comparison of the performance of monolayer MoS_2 FETs in our work with that in previous works is also summarized in Supplementary Table 2. From the comparison, the reliability of F-mica-assisted metal electrode transfer at large-scale can be clearly found. The array of 2D device fabricated via our method shows an enhanced averaged on/off ratio.

Supplementary Figures

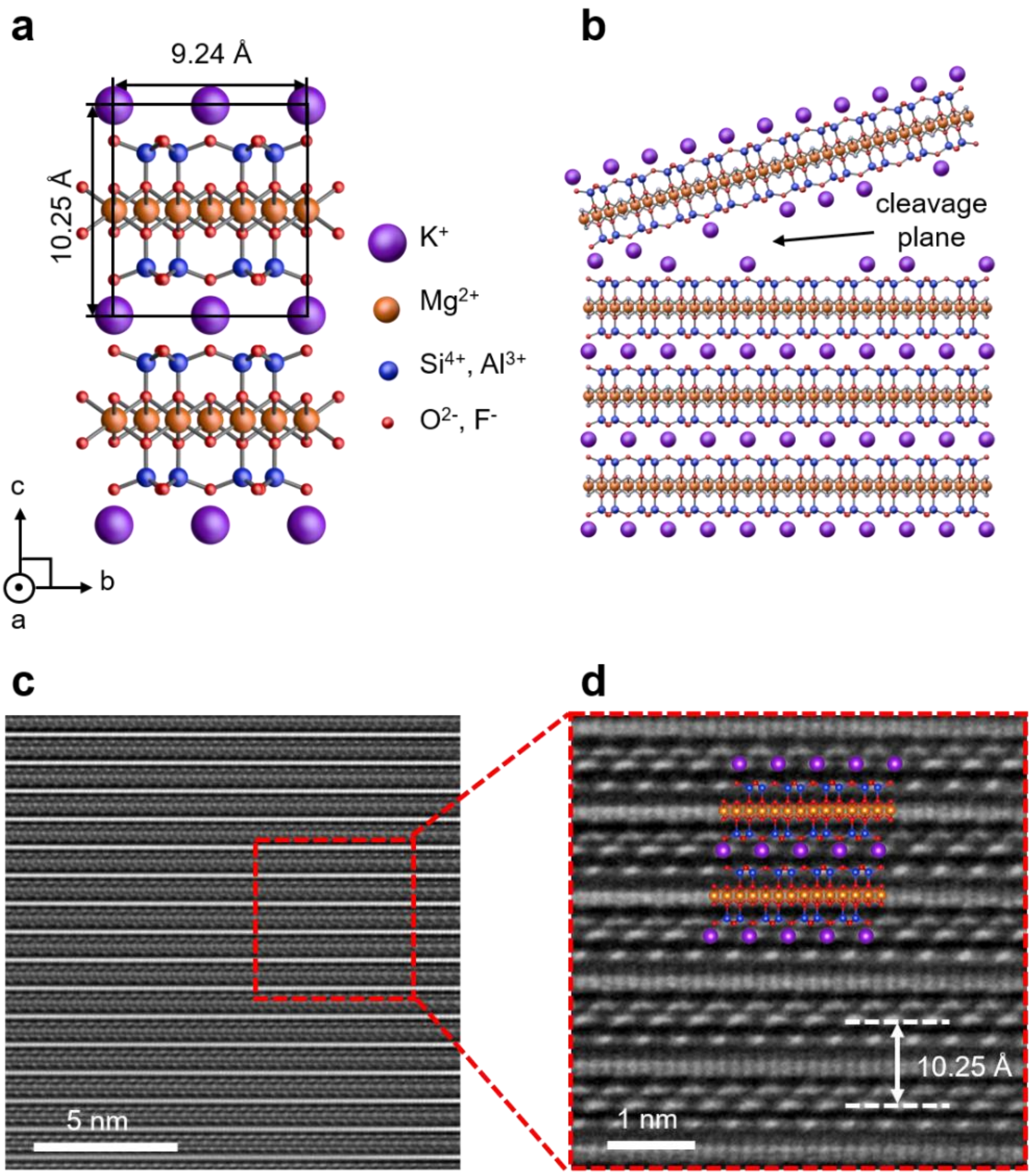

Supplementary Fig. 1: Crystal structure of F-mica. a Structure diagram of F-mica. **b** Schematic diagram of cleavage F-mica. Cleavage of F-mica along the K^+ ions layer produces two large and atomically flat surfaces with equal but randomly distributed K^+ ions preserving charge neutrality. **c** Cross-section STEM image of F-mica. **d** Zoomed STEM image of the red dashed box in **c**.

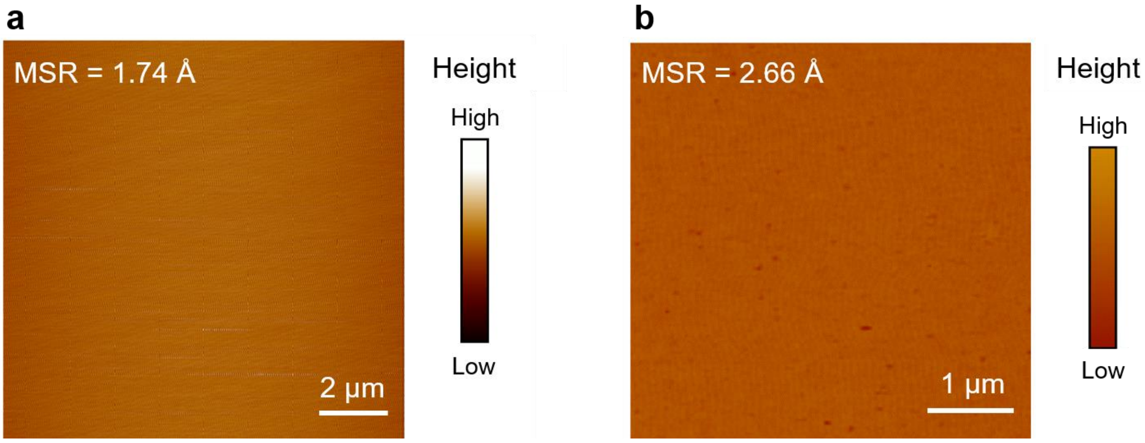

Supplementary Fig: 2. Surface topography of F-mica and peeled Au electrodes. a AFM image of fresh-peeled F-mica surface. **b** AFM image of the bottom side of the Au electrodes peeled from F-mica. The morphological surface roughness of the F-mica and the bottom side of Au electrode are 1.74 Å and 2.66 Å, respectively.

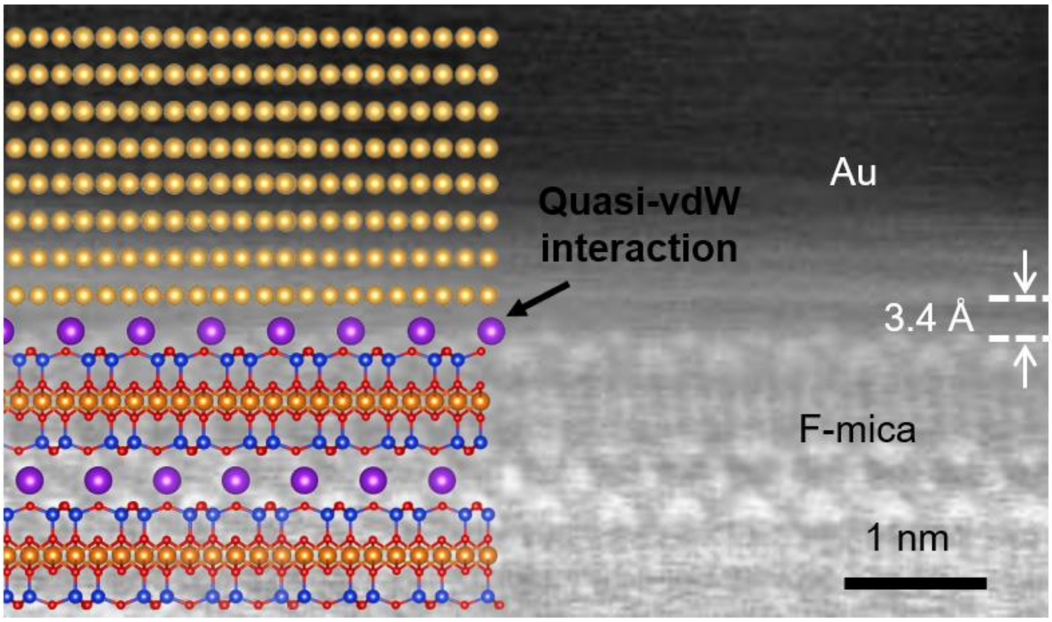

Supplementary Fig: 3. Cross-sectional STEM image of Au/F-mica interface. K^+ ions are intercalated between Au and F-mica. The gap of the Au/F-mica is ~ 3.4 Å.

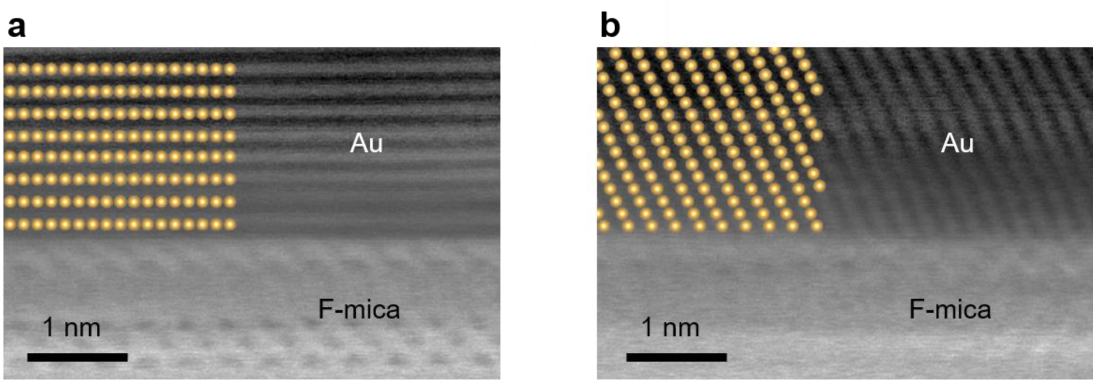

Supplementary Fig: 4. **Cross-sectional STEM images of Au/F-mica interface.** The deposited Au metals have different crystal orientations as shown in **a** and **b.**

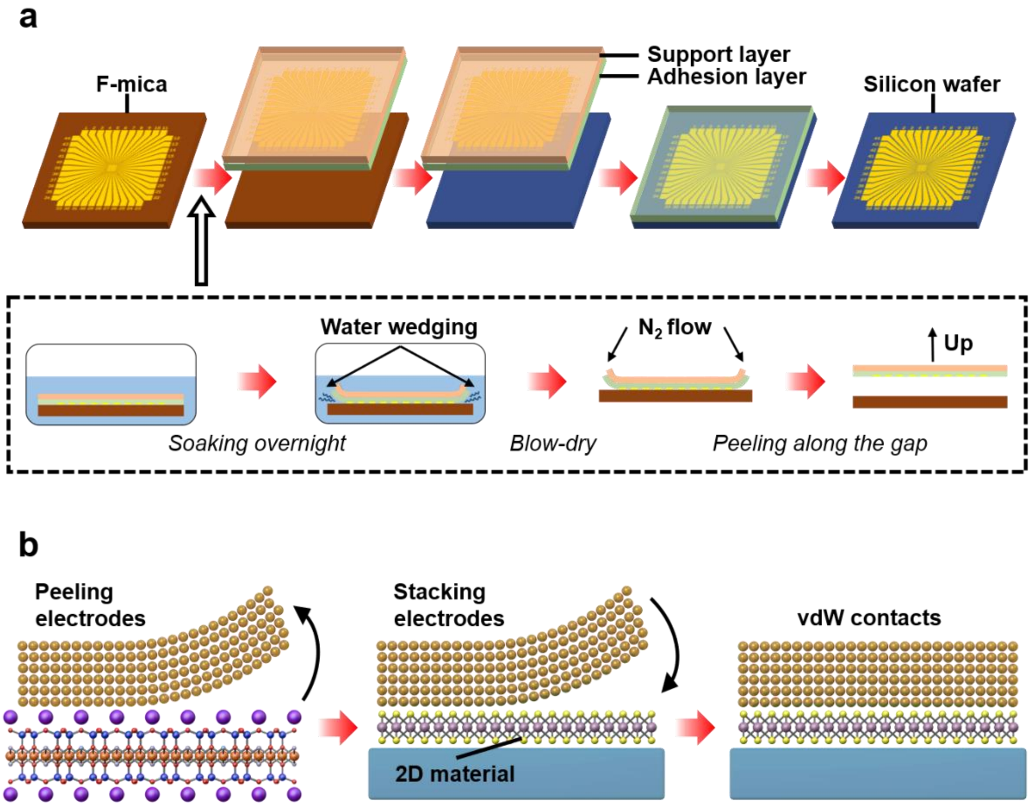

Supplementary Fig: 5. Schematic of electrodes transferring via F-mica. a Flow diagram of the electrodes transferring process via F-mica. Utilizing the hydrophilic property of F-mica, a tiny gap between the AL and the F-mica will be created at the edges after soaking in water overnight, which facilitates the AL and electrodes to be easily peeled off integrally. **b** Cross-sectional schematic of transferring metal electrodes from F-mica onto 2D materials.

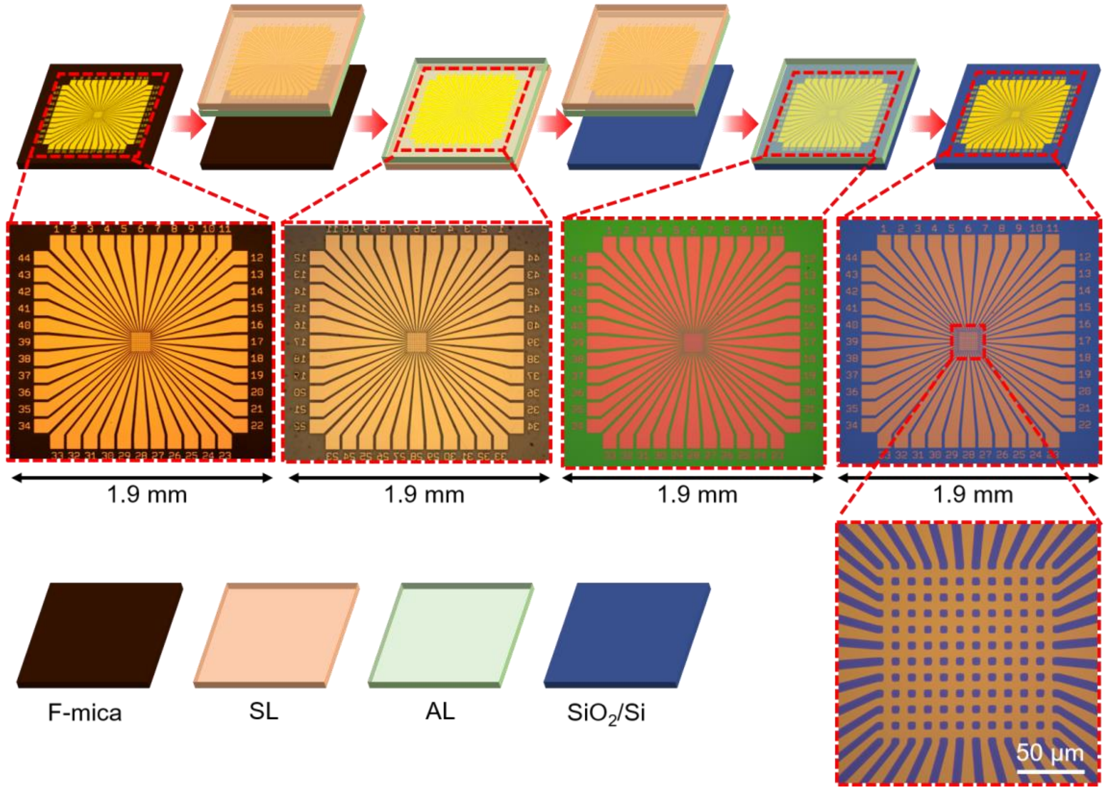

Supplementary Fig: 6. Transferring process for patterned Au electrodes. The whole patterned Au electrodes keep intact and unwrinkled after the transferring process.

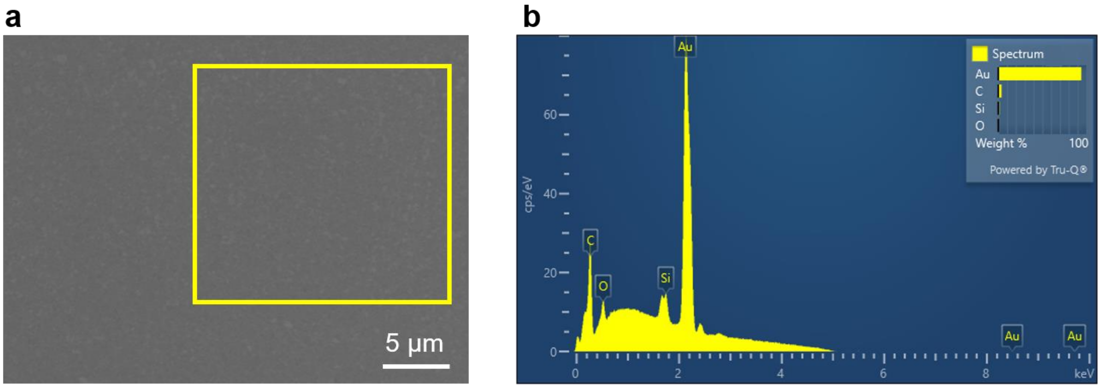

Supplementary Fig. 7: SEM image and selected-area EDS measurement of the peeled Au electrodes. a SEM image of the bottom side of the peeled Au electrodes on PDMS. **b** EDS measurement of the yellow boxed area in **a**. The detected elements are Au, Si, C, O in EDS analysis, without signal of Al, Mg, K, F from F-mica, which proves that no chemical doping from F-mica in transferring process. Besides, the C, O signal results from the adsorption air, and Si signal is derived from the PDMS substrate.

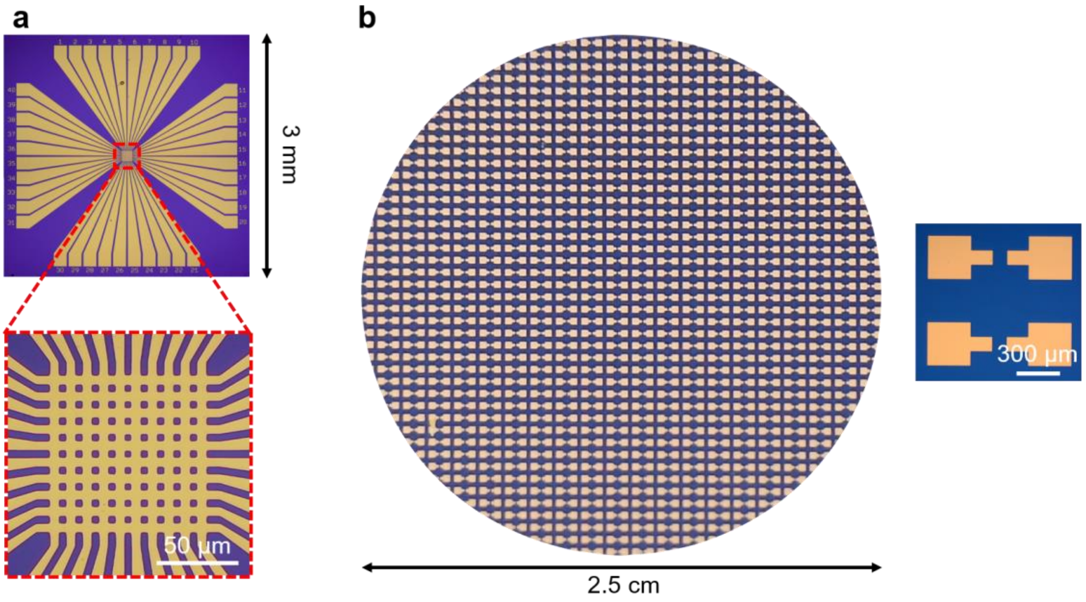

Supplementary Fig. 8: Demonstration of large-scale electrode transfer. a Optical image of stacked patterned Au electrodes (3 mm) on silicon substrate. The whole electrode pattern remains intact and unwrinkled after the transferring process. **b** Transferred Au electrode array on 1-inch Si wafer. Inset shows the zoomed optical image of the electrodes.

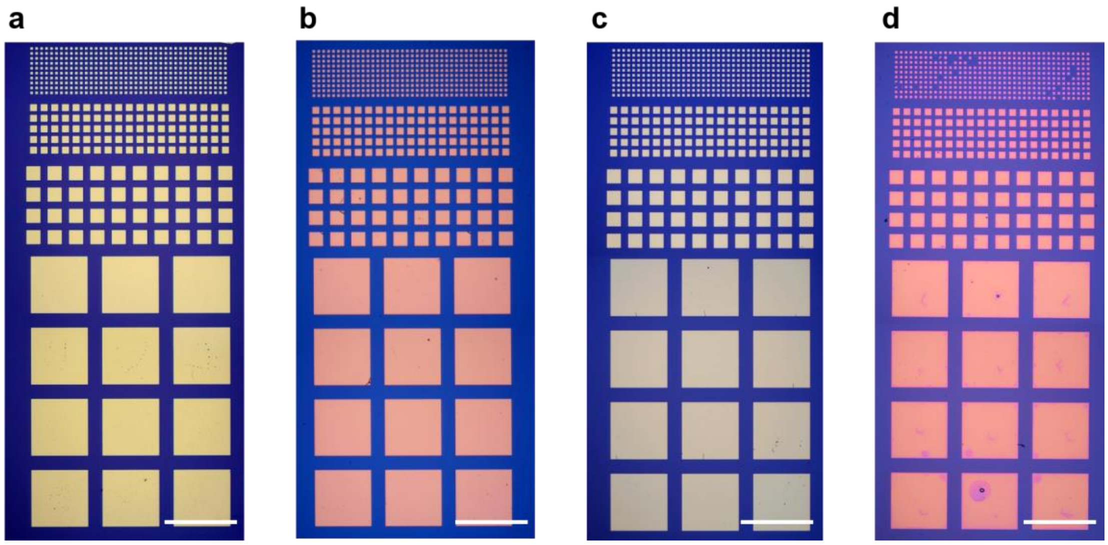

Supplementary Fig. 9: Demonstration of electrode transfer for different metals. a-d Optical images of Ag (**a**), Au (**b**), Pd (**c**) and Cu (**d**) pattern transferred on SiO_2/Si substrate via peeling from F-mica. All metal patterns are covered with 40 nm thick Au. The scale bar is 500 μm.

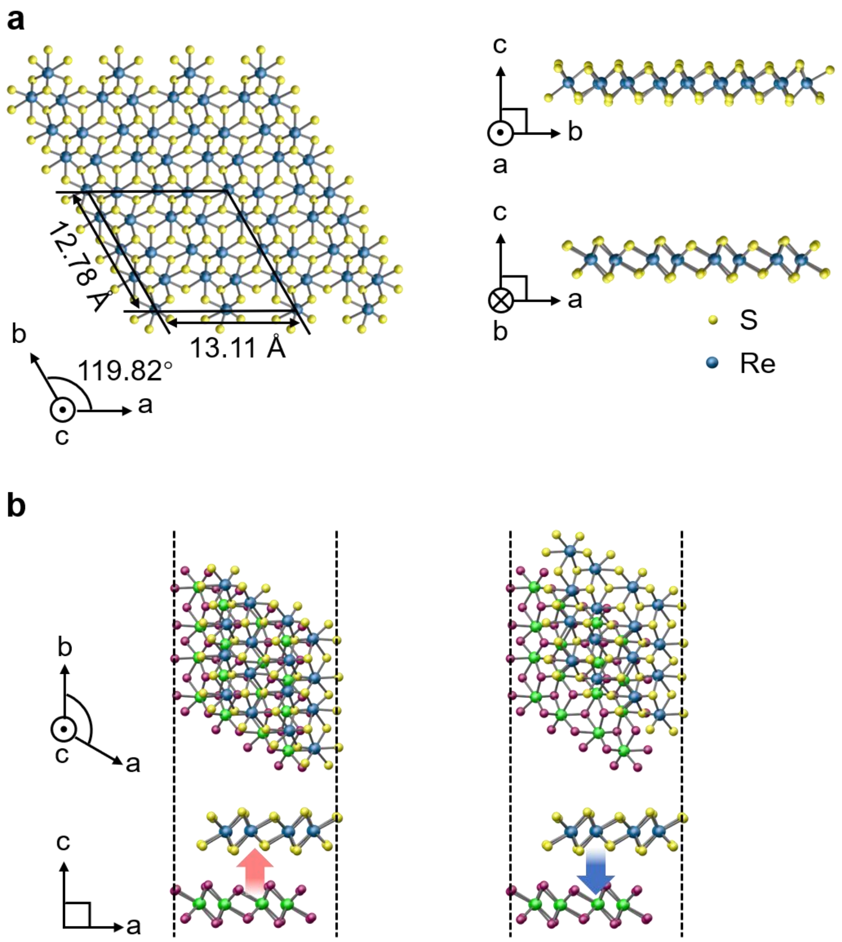

Supplementary Fig. 10: Lattice structure of 1T'-ReS_2. a Structure of monolayer 1T'-ReS_2. **b** Top and side views of bilayer ReS_2 with two polarization states. The red and blue arrows indicate the upward and downward polarization directions, respectively.

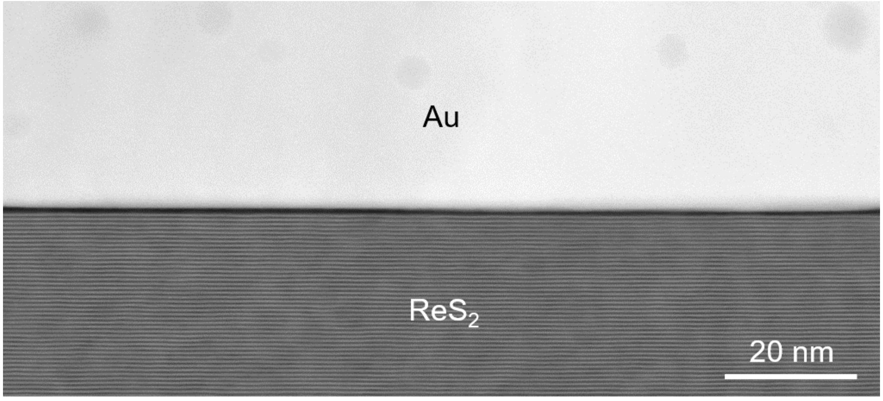

Supplementary Fig. 11: Cross-sectional STEM image of the stacked Au/ReS_2 structure. A clear and clean vdW interface is observed between Au and ReS_2.

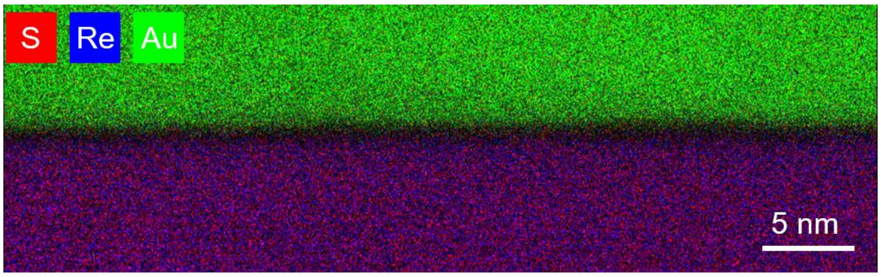

Supplementary Fig. 12: Elemental distribution of the stacked Au/ReS_2 structure. There is an apparent interface gap without the existence of any elements.

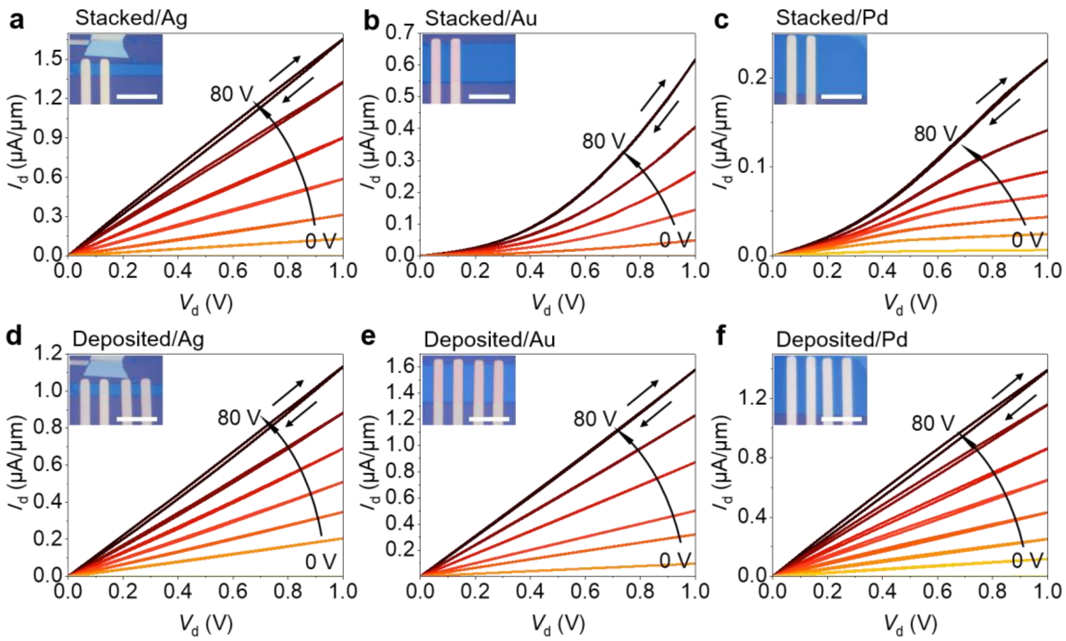

Supplementary Fig. 13: *I-V* measurements of the ReS_2 FETs in Fig. 2. a-c Output characteristic curves of ReS_2 FETs with Ag (**a**), Au (**b**), and Pd (**c**) electrodes by stacking. **d-f** Output characteristic curves of ReS_2 FETs with Ag (**d**), Au (**e**), and Pd (**f**) electrodes by deposition. The insets are optical images of these devices. The scale bar in the images is 20 μm.

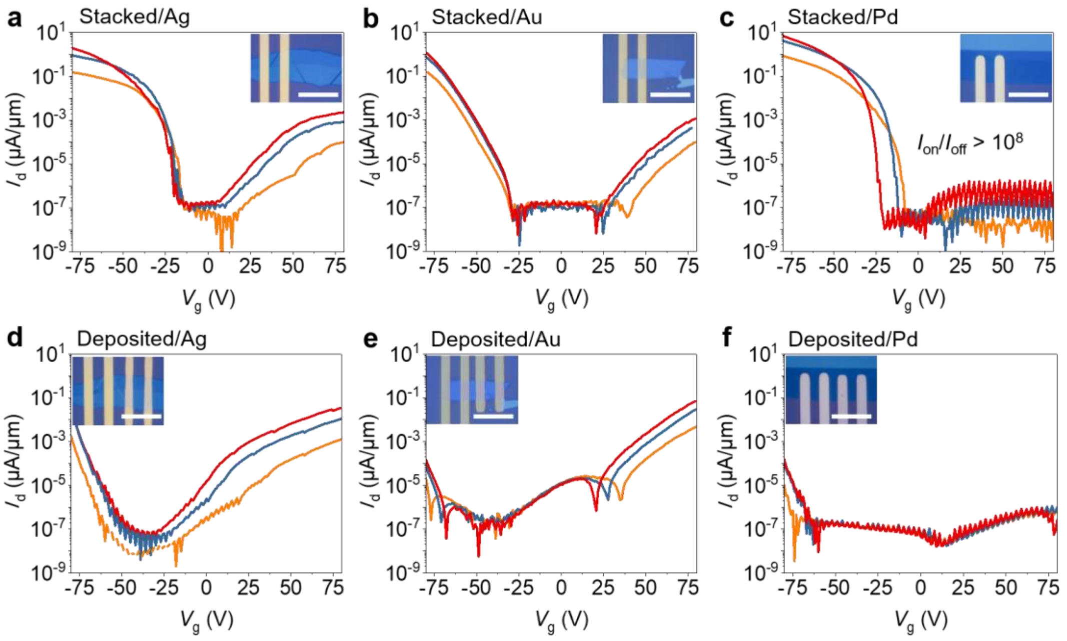

Supplementary Fig. 14: Electrical properties of WSe_2 FETs with stacked and deposited metal electrodes. a-c Transfer characteristic curves of WSe_2 FETs with Ag (**a**), Au (**b**), and Pd (**c**) electrodes by stacking. **d-f** Transfer characteristic curves of WSe_2 FETs with Ag (**d**), Au (**e**), and Pd (**f**) electrodes by deposition. The WSe_2 FETs in **d-f** use the corresponding samples in **a-c**, respectively. The red, blue and orange curves in **a-f** are measured under drain voltage of 1 V, 0.5 V and 0.1 V, respectively. The insets are optical images of the measured devices. The scale bar is 20 μm.

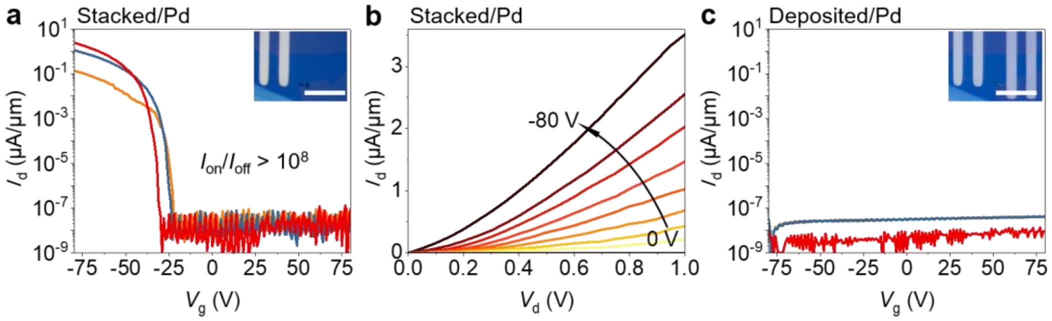

Supplementary Fig. 15: Electrical properties of monolayer WSe_2 FETs with stacked and deposited Pd electrodes. a Transfer characteristic curves of monolayer WSe_2 FET with stacked Pd electrodes. **b** Output characteristic curves of the FET in **a** under various V_g from 0 to -80 V. **c** Transfer characteristic curves of monolayer WSe_2 FET with deposited Pd electrodes. The WSe_2 flake in **c** is the same sample from **a**. The red, blue, and orange curves in **a** and **c** are measured under drain voltage of 1, 0.5, and 0.1 V, respectively. The insets are optical images of measured devices. The scale bar is 20 μm.

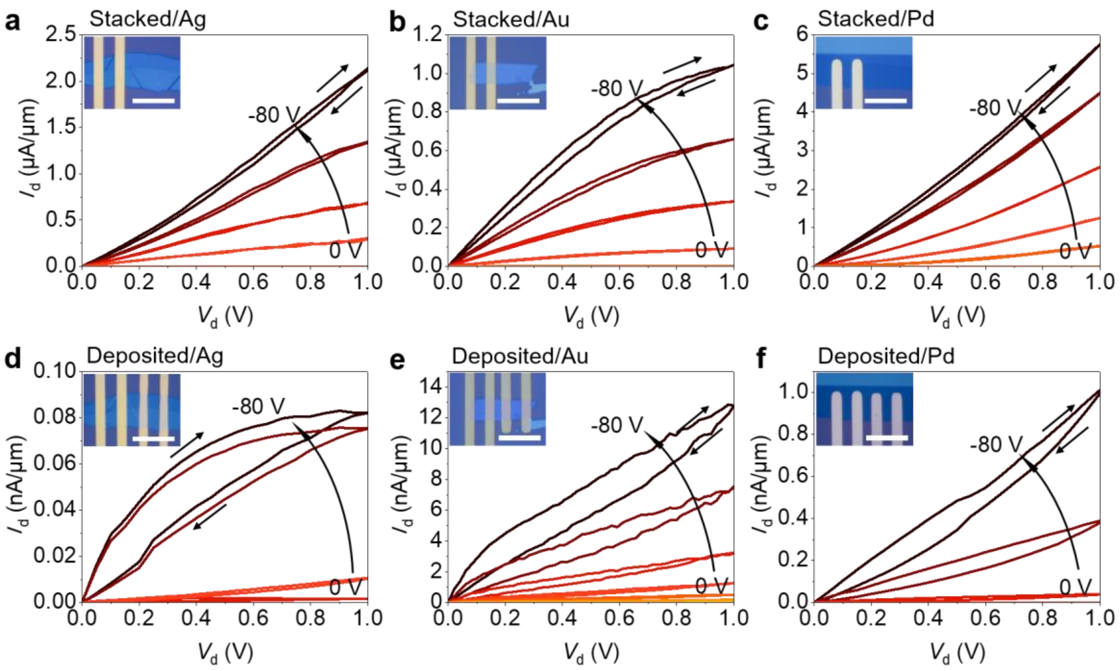

Supplementary Fig. 16: *I*-*V* measurements of the WSe_2 FETs in Supplementary Fig. 14. a-c Output characteristic curves of WSe_2 FETs with Ag (**a**), Au (**b**), and Pd (**c**) electrodes by stacking. **d-f** Output characteristic curves of WSe_2 FETs with Ag (**d**), Au (**e**), and Pd (**f**) electrodes by deposition. The insets are optical images of the measured devices. The scale bar is 20 μm.

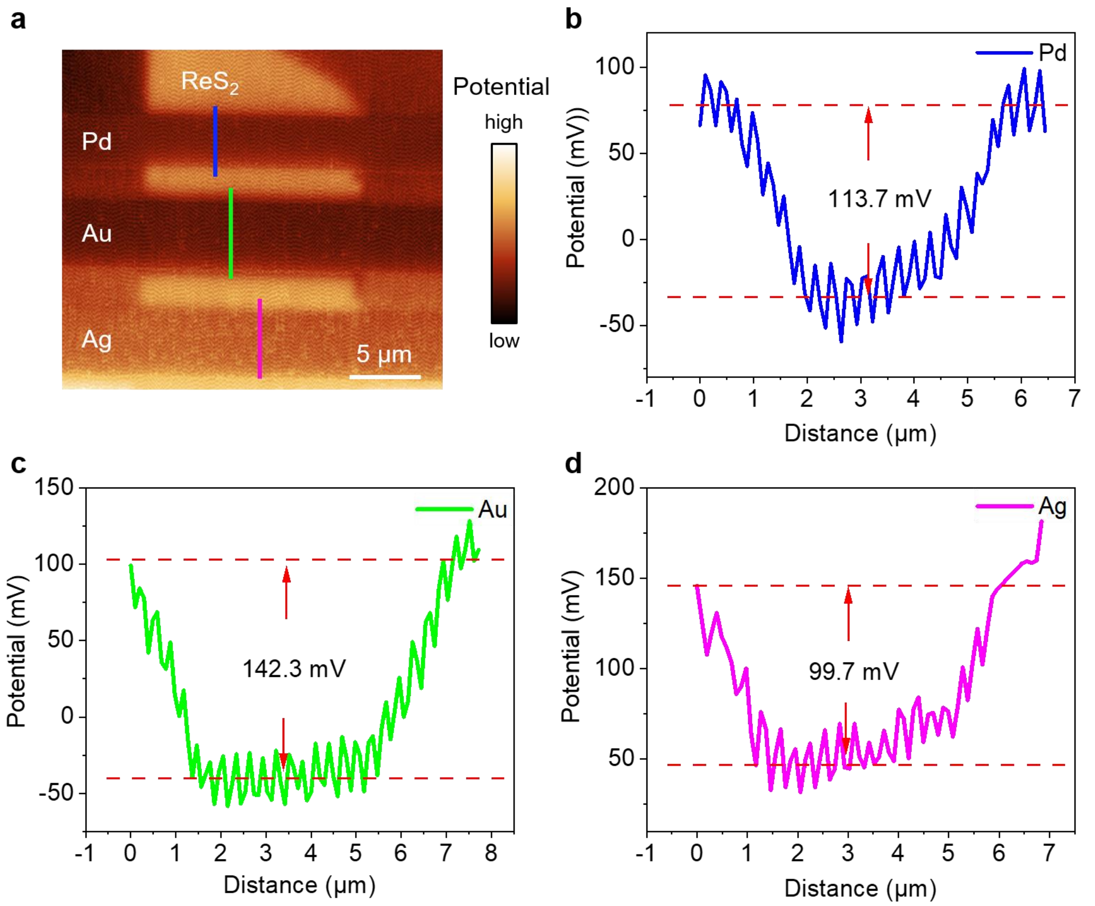

Supplementary Fig. 17: Surface potential of stacked electrodes. a Surface potential of stacked Pd, Au, and Ag electrodes on the same ReS_2 flake. **b-d** Surface potential differences along the line profiles in **a** for Pd (blue), Au (green), and Ag (pink) electrodes.

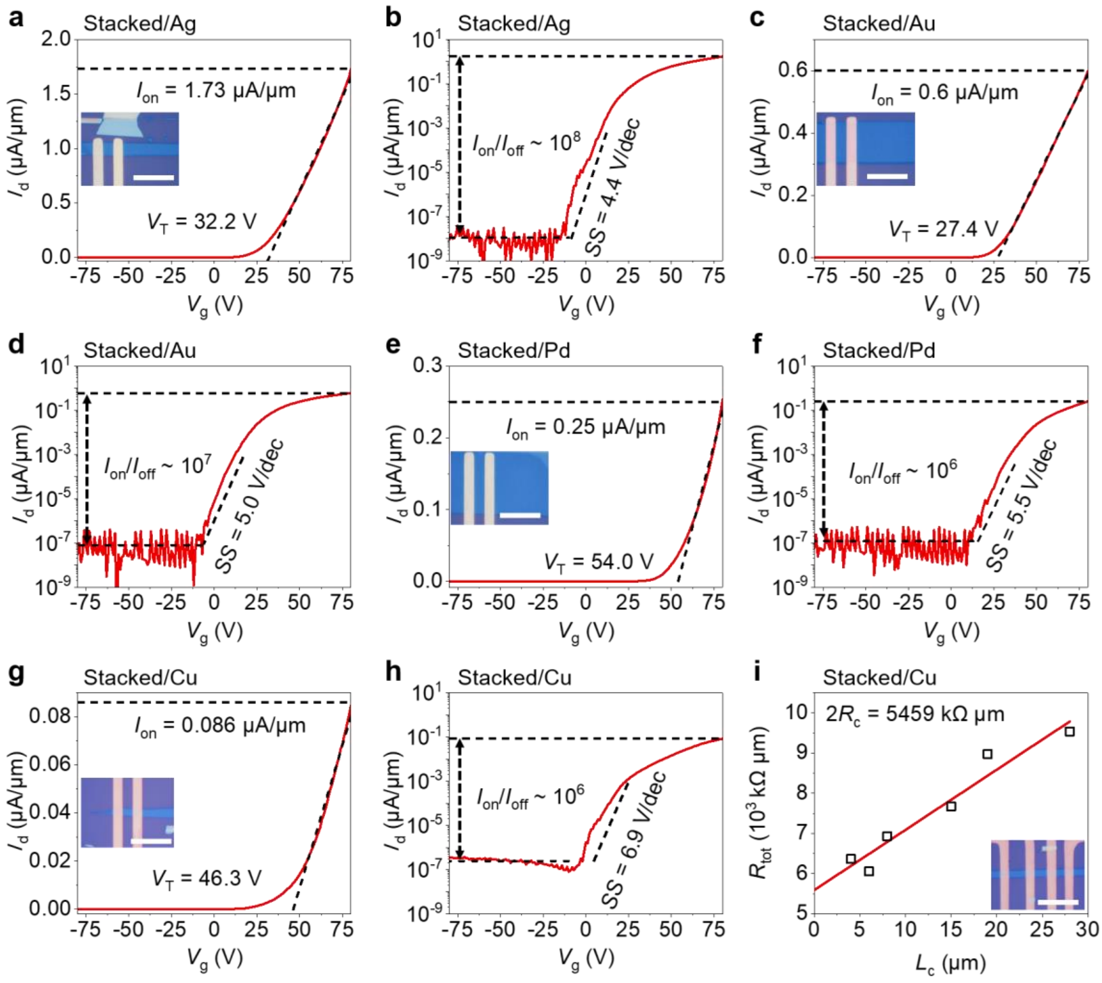

Supplementary Fig. 18: Electrical properties of ReS_2 FETs with stacked metal electrodes. a-h Transfer characteristic curves of ReS_2 FETs with stacked Ag (**a-b**), Au (**c-d**), Pd (**e-f**), and Cu (**g-h**) electrodes. The applied bias is 1 V. **i** Contact resistance of ReS_2 FETs with Cu electrodes by stacking. The insets are optical images of the measured devices. The scale bar is 20 μm.

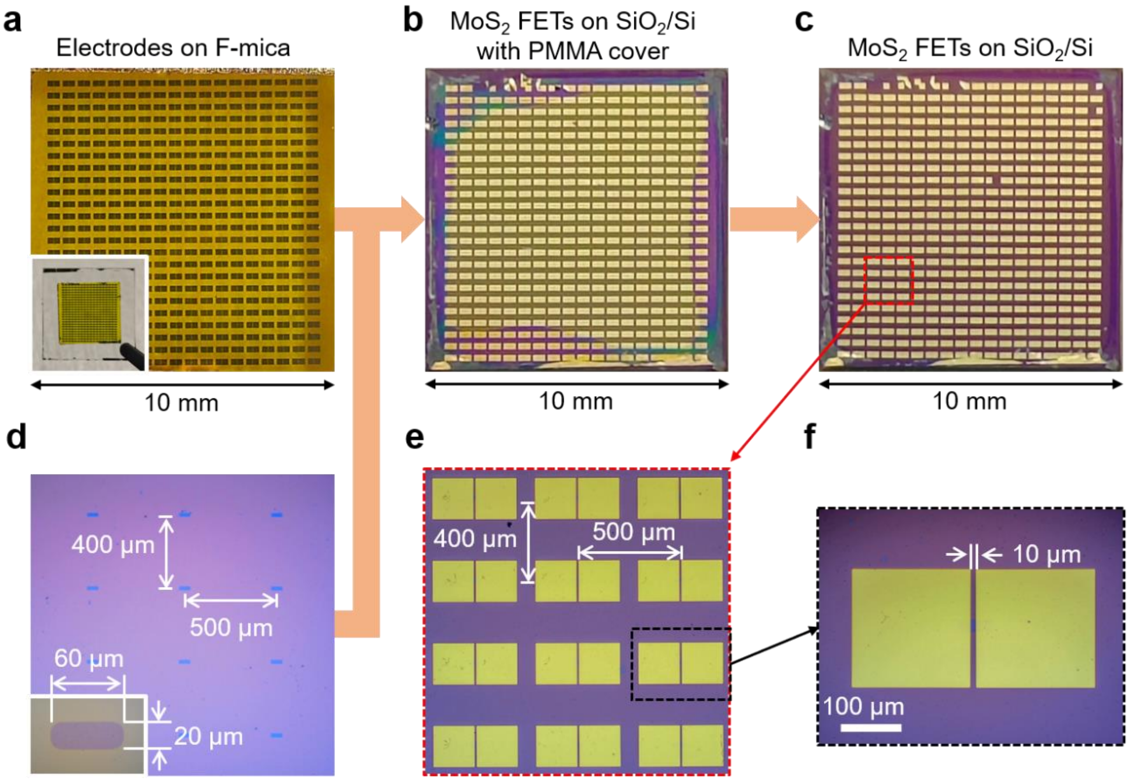

Supplementary Fig. 19: Fabrication process of ML MoS_2 FET array at 10 × 10 mm^2. a Optical image of the Ag/Au electrodes deposited on F-mica. **b**, **c** Optical images of the ML MoS_2 FET array with transferred Ag/Au electrodes shown in **a** on SiO_2/Si before (**b**) and after (**c**) dissolving adhesion layer (PMMA). **d** Zoomed optical image of ML MoS_2 ribbon array on SiO_2/Si used as the channels of FETs in **b** and **c**. **e-f** Zoomed optical images of the red and black dashed regions in **c** and **e**.

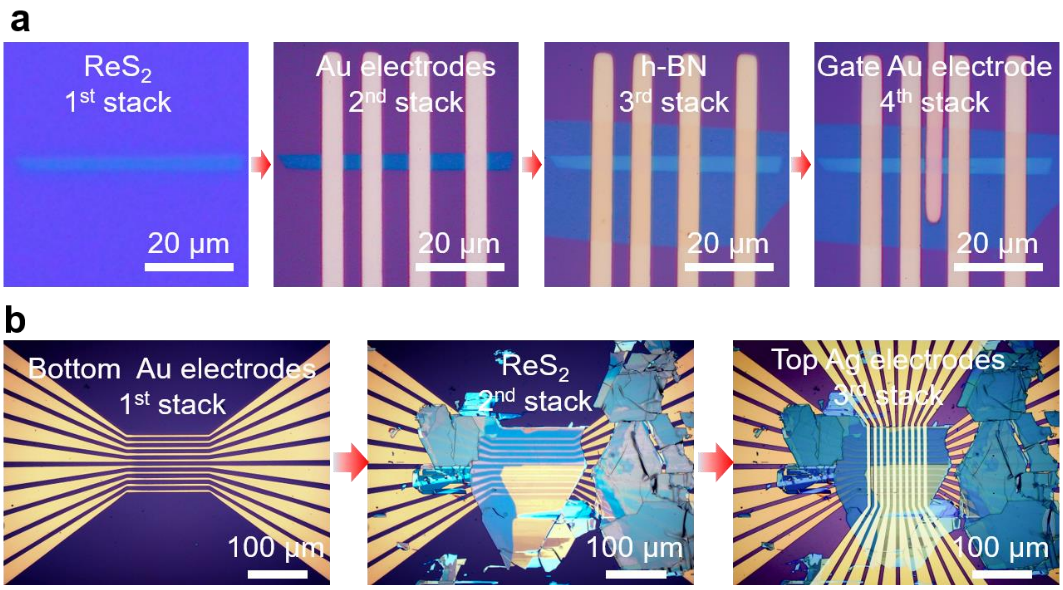

Supplementary Fig. 20: Fabricating process of 2D devices with multiple layer structure. a Fabrication process of the top-gate ReS_2 FET in **Fig. 4b**. **b** Fabrication process of the Ag/ReS_2/Au FTJ array in **Fig. 4e**.

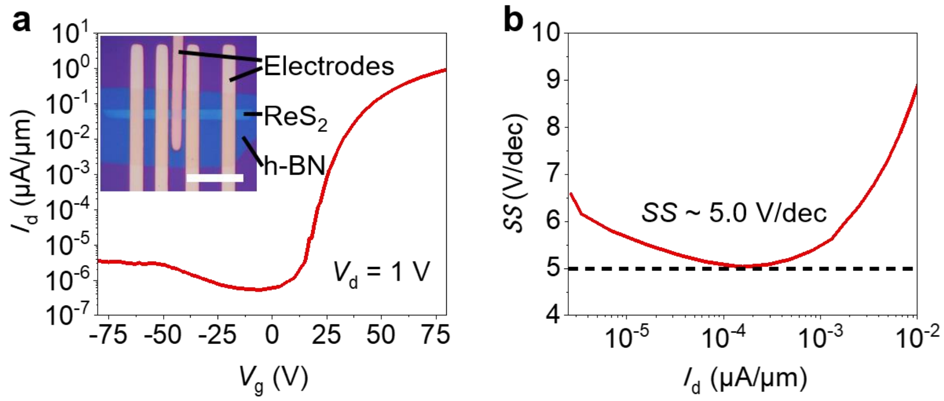

Supplementary Fig. 21: Electrical properties of ReS_2 FET in Fig. 4b. a Transfer characteristic curve of the ReS_2 FET in **Fig. 4b** at 1 V bias. **b** *SS* values of the ReS_2 FET at different current densities in **a**.

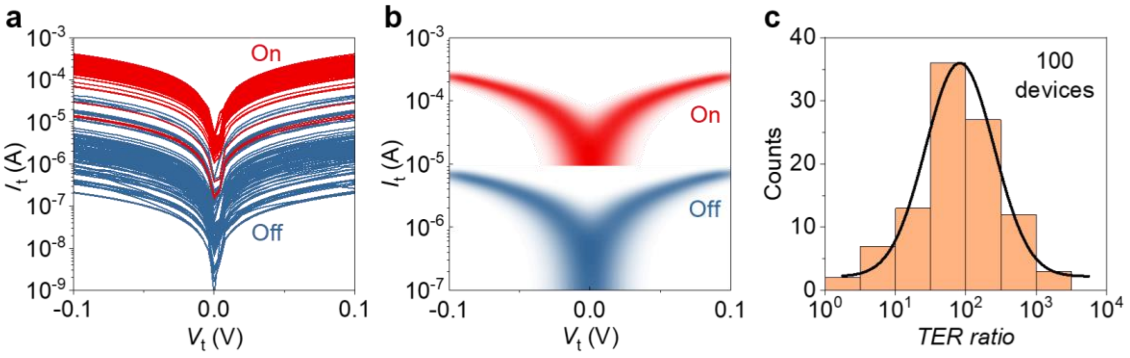

Supplementary Fig. 22: Electrical properties of 100 ReS_2 FTJs in Fig. 4e. a Off-state and on-state *I*-*V* curves of the100 Ag/ReS_2/Au FTJs in **Fig. 4e**. **b** The current density distribution of the *I*-*V* curves in **a**. The darker the color, the higher the density. **c** The statistics of the TER ratios of the 100 Ag/ReS_2/Au FTJs in **Fig. 4e**.

Supplementary Tables

P-type characteristic of monolayer WSe_2 FETs						
Method	L_{ch} (μm)	Oxide layer	V_{ds} (V)	I_{on} (μA/μm)	On/off ratio	Ref.
**Stacking electrodes via F-mica (Pd/Au)**	**5**	**SiO_2**	**1**	**3.5**	**10^8**	**This work**
Low-temperature depositing electrodes (Pt)	1.5	SAMs/ SiO_2	1	7.6	10^6	*Nature* 610, 61–66 (2022)
Stacking electrodes via Si wafer (Au)	5	SiO_2	1	0.48		*Nat. Commun.* 11, 1866 (2020)
Se buffer layer (Au)	2	h-BN /SiO_2	1	< 3	10^5	*Nat. Electron.* 5, 241–247 (2022)
Stacking electrodes via h-BN (Pt)	9	h-BN /SiO_2	0.1	0.4	10^4	*Nat. Electron.* 2, 187–194 (2019)
Deposited electrodes (Pt)	5	Al_2O_3 /HZO	1	~ 1	10^5	*64th IEDM*, 22.3.1–22.3.4 (2018)
Deposited electrodes with/o NO_2 doping (Pd/Au)	9.4	SiO_2	1	0.005 (undoped) 7 (doped)	10^3 (undoped) 10^6 (doped)	*Nano Lett.* 12, 3788-3792 (2012)
Deposited electrodes (Ti/Pd)	3	SiO_2	5	~ 0.08	10^5	*ACS Nano* 9, 6119-6127 (2015)

L_{ch}, channel length; V_d, drain voltage; I_{on}, on-state current; SAMs, self-assembled monolayers.

Supplementary Table 1. P-type characteristic of monolayer WSe_2 FETs.

Performance of CVD-grown monolayer MoS_2 FETs								
Method	L_{ch} (μm)	Oxide layer	V_{ds} (V)	Average I_{on} (μA/μm)	Average On/off ratio (10^6)	Number of devices	Yield (%)	Ref.
**Stacking electrodes via F-mica (Ag/Au)**	**10**	**SiO_2**	**1**	**0.14**	**6.8**	**396**	**96.3**	**This work**
Stacking electrodes via graphene (Ag/Au)	15	SiO_2	1	0.109	4.8	100		*Nat. Electron.* 5, 275–280 (2022)
Stacking electrodes via graphene (Au)		Bottom: SiO_2 Top: Y_2O_3	0.5			18	70	*Nat. Nanotechnol.* 17, 1206–1213 (2022)
Stacking electrodes via Si wafer (Ag/Au)	6	Al_2O_3	6	16	11	60		*Nat. Nanotechnol.* 18, 471–478 (2023)
Deposited electrodes (Ti/Au/Ti)	5-60	Bottom: Al_2O_3 Top: HfO_2 or Al_2O_3			1	100	96	*Nano Res.* 16, 12794-12799 (2023)
Deposited electrodes (Au)	10	Bottom:a-Al_2O_3 Top: HfO_2	1		~ 100	333		*Nat. Mater.* 22, 1324-1331 (2023)

L_{ch}, channel length; V_d, drain voltage; I_{on}, on-state current.

Supplementary Table 2. Performance of CVD-grown monolayer MoS_2 FETs.

Supplementary References

1. Molina-Mendoza, A. J. *et al.* Centimeter-scale synthesis of ultrathin layered MoO_3 by van der Waals epitaxy. *Chem. Mater.* **28**, 4042–4051 (2016).

2. Castellanos-Gomez, A. *et al.* Deterministic transfer of two-dimensional materials by all-dry viscoelastic stamping. *2D Mater.* **1**, 011002 (2014).

3. Wan, Y. *et al.* Room-temperature ferroelectricity in 1T'-ReS_2 multilayers. *Phys. Rev. Lett.* **128**, 067601 (2022).

4. Liu, Y. *et al.* Approaching the Schottky–Mott limit in van der Waals metal-semiconductor junctions. *Nature* **557**, 696–700 (2018).

5. Kong, L. *et al.* Doping-free complementary WSe_2 circuit via van der Waals metal integration. *Nat. Commun.* **11**, 1866 (2020).

6. Yen, M., Bitla, Y. & Chu, Y.-H. van der Waals heteroepitaxy on muscovite. *Materials Chemistry and Physics* **234**, 185–195 (2019).

7. Kwon, G. *et al.* Interaction- and defect-free van der Waals contacts between metals and two-dimensional semiconductors. *Nat. Electron.* **5**, 241–247 (2022).

8. Kong, L. *et al.* Wafer-scale and universal van der Waals metal semiconductor contact. *Nat. Commun.* **14**, 1014 (2023).

9. Liu, G. *et al.* Graphene-assisted metal transfer printing for wafer-scale integration of metal electrodes and two-dimensional materials. *Nat. Electron.* **5**, 275–280 (2022).

10. Yang, X. *et al.* Highly reproducible van der Waals integration of two-dimensional electronics on the wafer scale. *Nat. Nanotechnol.* **18**, 471–478 (2023).
